# Next-Generation MDMA Analogue SDMA: Pharmacological and Metabolic Insights

**DOI:** 10.1021/acschemneuro.5c00782

**Published:** 2025-12-02

**Authors:** Nina Kastner, Núria Nadal-Gratacós, Selina Hemmer, Leticia Alves da Silva, John L. McKee, Tamara Hell, Giulia Cicalese, Marion Holy, Fatemeh Kooti, Kathrin Jäntsch, Ricarda Baron, Naomi Shacham, Bruna Cuccurazzu, Adam L. Halberstadt, John D. McCorvy, Thomas Stockner, Markus R. Meyer, Raúl López-Arnau, Matthias Grill, Harald H. Sitte

**Affiliations:** 1 Institute of Pharmacology, Center for Physiology and Pharmacology, 27271 Medical University of Vienna, Vienna 1090, Austria; 2 Department of Pharmacology, Toxicology and Therapeutic Chemistry, Faculty of Pharmacy, Pharmacology Section and Institute of Biomedicine (IBUB), 16724 University of Barcelona, Barcelona 08028, Spain; 3 Experimental and Clinical Toxicology and Pharmacology, Center for Molecular Signaling (PZMS), PharmaScienceHub (PSH), Saarland University, Homburg 66424, Germany; 4 Department of Cell Biology, Neurobiology and Anatomy, 5506 Medical College of Wisconsin, Milwaukee, Wisconsin 53226, United States; 5 MiHKAL GmbH, Stansstad 6362, Switzerland; 6 Department of Psychiatry, 8784 University of California San Diego, 9500 Gilman Drive, La Jolla, California 92093-0021, United States; 7 Department of Pharmacology and Toxicology, Neuroscience Research Center, 5506 Medical College of Wisconsin, Milwaukee, Wisconsin 53226, United States; 8 Hourani Center for Applied Scientific Research, Al-Ahliyya Amman University, 19328 Amman, Jordan; 9 AddRess Centre for Addiction Research and Science, 27271 Medical University of Vienna, 1090 Vienna, Austria

**Keywords:** MDMA, analogues, monoamine transporters, monoamine receptors, pharmacological profile, hepatic metabolism

## Abstract

3,4-Methylenedioxymethamphetamine (MDMA), commonly known as ecstasy, shows promise in treating depression and post-traumatic stress disorder (PTSD), resulting in breakthrough status. However, concerns regarding MDMA’s abuse potential and cytotoxicity have sparked interest in developing safer analogues with similar therapeutic benefits. This study investigated the pharmacological properties of MDMA analogues in which the 1,3-benzodioxole group is replaced by a 1,3-benzoxathiole, termed SDA and SDMA, compared to MDA and MDMA through *in silico*, *in vitro*, and *in vivo* assays. *In vitro* experiments using human embryonic kidney (HEK293) cells examined the interactions with monoamine transporters. SDA and SDMA showed similar profiles to MDMA at the serotonin transporter (SERT), while both inhibited dopamine (DAT) and norepinephrine (NET) transporters more potently, in line with *in silico* molecular docking fitness scores of binding. SDA and SDMA also showed increased potency in evoking efflux through SERT and DAT acting as partial releasers. SDA and SDMA exhibited a similar interaction profile with 5-HT_2_ receptors compared with their respective analogues. Metabolism studies revealed faster clearance rates for SDA and SDMA, in contrast to MDA and MDMA, which exhibited only weak degradation. In contrast to MDMA’s rewarding effects, SDMA did not induce significant effects in mice, while SDA only produced a significant preference for the drug-paired compartment at the lowest dose tested. Moreover, while SDMA shares similar locomotor and hyperthermic profiles as MDMA in mice, SDA induced increased hyperlocomotion and more sustained hyperthermia. In conclusion, these findings suggest that SDMA, with enhanced metabolic profiles and reduced abuse potential, is a promising candidate for further studies.

## Graphical abstract

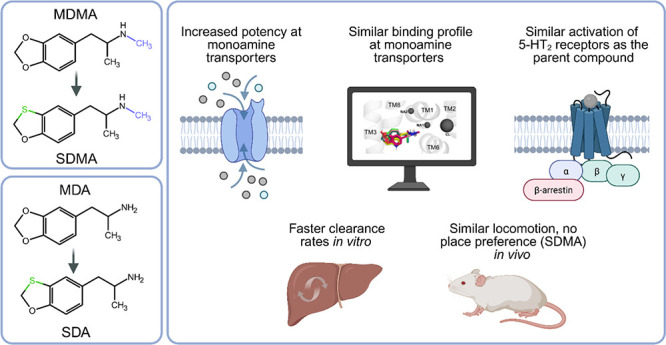

## Introduction

3,4-Methylenedioxymethamphetamine (MDMA), also known as ecstasy, is a commonly used recreational drug, which has regained research interest over the past two decades. Due to its psychoactive effects, it became popular in the late 1970s, followed by its regulation as a scheduled substance in the 1980s.^1^ Clinically, acute effects of MDMA include an enhanced mood, mild dissociation from reality, pro-social effects, and increased empathy for others (“entactogenic effects”).^2^ Therefore, MDMA is mainly categorized as an entactogen or empathogen.^3^ However, recently, some authors grouped MDMA as a psychedelic drug based on its serotonin (5-HT) 2A (5-HT_2A_) receptor agonism,^4,5^ though this classification remains controversial. Pharmacologically, MDMA acts as a substrate at the monoamine transporters (MATs) of the solute-carrier 6 (SLC6) family for dopamine (DA), norepinephrine (NE), and 5-HT, evoking nonexocytotic efflux of the respective neurotransmitters.^6,7^ It further acts at the vesicular monoamine transporter 2 (VMAT2) as a substrate-like releaser,^8^ thereby causing an increase of neurotransmitter concentrations in the cytoplasm of presynaptic neurons.^9,10^ It was also reported that MDMA interacts with the 5-HT_2_ receptor family, which are believed to be at least partially responsible for its psychoactive effects.^11–14^

Most recently, a number of clinical observations have sparked interest in MDMA as an essential therapeutic adjunct treatment for psychotherapy,^15–17^ especially in addressing post-traumatic stress disorder.^18,19^ These promising outcomes observed in clinical studies have led to the approval of MDMA in Australia, and it is anticipated that it will soon gain approval as an official treatment in additional countries.^20^ However, the United States Food and Drug Administration (FDA) recently voted against the approval of MDMA based on several issues, first raised by members of a scientific review panel including deficiencies with declaring conflicts of interest, phase 2 clinical trials, effectiveness of patient blinding, and the potential for toxicity and abuse liability.^21–95^ The panel suggested that the latter issues can potentially be addressed by investigating similar MDMA analogues to identify important features to retain therapeutic potential but also minimize adverse events.^22^

However, MDMA may be contraindicated for patients who do not respond well to MDMA’s specific mode of action such as individuals with pre-existing cardiovascular conditions or a history of extensive stimulant drug use, and there is limited availability of alternatives to MDMA. MDMA use can cause mydriasis, jaw clenching, anorexia, increased or decreased anxiety, and hyperthermia.^23,24^ In clinical settings, MDMA is administered at low doses, as there is evidence that high doses of MDMA can damage serotonergic neurons due to oxidative stress and/or inflammation and lead to a decrease in 5-HT termini^25–27^ and a gradual breakdown of the serotonergic neurotransmission system.^28^ However, MDMA metabolites such as catechol and quinone metabolites may also possibly contribute to the sustained neurotoxic effects due to the production of free radicals.^28–31^ Direct intracerebroventricular administration of MDMA results in significantly reduced neurotoxicity, providing strong evidence that metabolites induce at least part of the neurotoxic effects.^32–34^ In general, the metabolism of MDMA follows nonlinear pharmacokinetics most likely due to the inhibition of cytochrome P450 enzymes, which is linked with the methylenedioxyphenyl group of MDMA.^28,35,36^

In this study, we investigated two novel analogues of MDMA and 3,4-methylendioxyamphetamine (MDA), which is an MDMA metabolite. The analogues replace the 1,3-benzodioxole (methylenedioxyphenyl) group with 1,3-benzoxathiole, resulting in 1-(1,3-benzoxathiol-5-yl)-*N*-methylpropan-2-amine (SDMA) and 1-(1,3-benzoxathiol-5-yl)­propan-2-amine (SDA). The aims of this study were to (i) characterize the *in vitro* pharmacological profile of the novel analogues at DA transporter (DAT), NE transporter (NET), 5-HT transporter (SERT), organic cation transporters (OCT) 1–2, and 5-HT_2_ receptor subtypes; (ii) investigate *in silico* binding poses at SERT, DAT, and NET; (iii) elucidate the *in vitro* hepatic metabolism in contrast to MDA and MDMA; and (iv) investigate *in vivo* behavioral and core body temperature change of novel analogues compared to MDMA. Through our investigation, we found that SDA and SDMA retain interactions for key pharmacological targets but point to a slightly lower abuse reward potential and higher metabolic clearance. These improvements make them a promising alternative to MDMA and address the concerns of the FDA panel.

## Results and Discussion

MDMA emerged as promising adjunctive treatment for PTSD, which led to its breakthrough status.^18,19^ Administration of MDMA supported by psychotherapy significantly improved outcomes for PTSD patients, while first-line treatments such as selective serotonin reuptake inhibitors (SSRIs) typically only reduce symptoms slightly and are completely ineffective for approximately 40% of patients.^37,38^ MDMA has subsequently been approved in Australia and New Zealand as an effective adjunctive treatment.^20^ However, the U.S. FDA recently declined the approval of MDMA. While concerns arose on an administrative level, there were also safety concerns regarding MDMA.^21^ Several studies have already investigated MDMA analogues,^39–42^ including a recent study from our laboratory,^22^ to explore potential approaches to improve the safety profile. Due to promising results in our earlier study,^22^ we investigated two novel analogues of MDMA and MDA termed SDMA and SDA that contain a replacement of the 1,3-benzodioxole (methylenedioxyphenyl) group with 1,3-benzoxathiole (Figure 1A).

**Figure 1:**
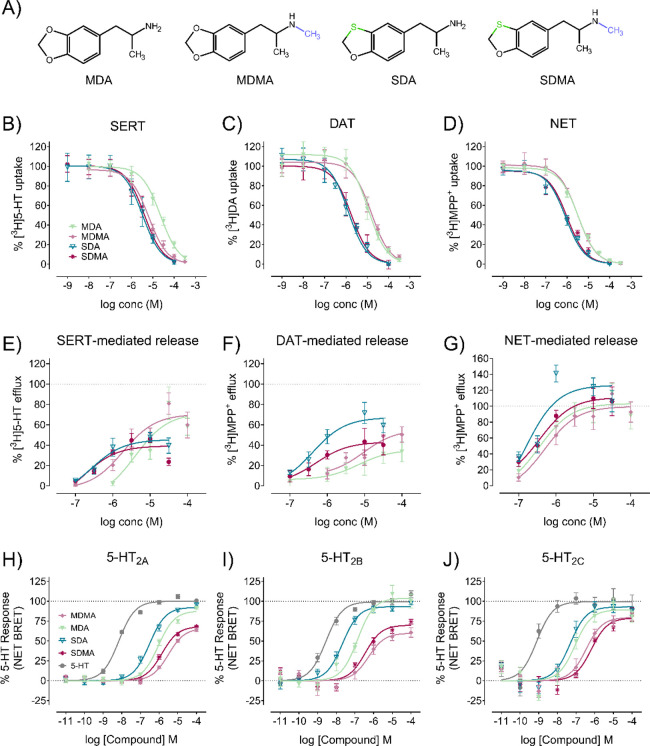
(A) Structures of MDA, MDMA, SDA, and SDMA. Uptake inhibition assays of substances at (B) SERT, (C) DAT, and (D) NET. Curves were fitted with a sigmoidal dose–response curve to obtain half-maximal inhibitory concentration (IC_50_) values (see Table 1). Transporter-mediated efflux of (E) SERT, (F) DAT, and (G) NET in a concentration dependent matter of the compound of interest is fitted with a sigmoidal dose–response curve to obtain half maximal effective concentration (EC_50_) values (see Table 1). Individual data points are represented with mean ± standard deviation (SD) from three to five independent experiments, each performed in triplicate. Effects of MDA, MDMA, SDA, and SDMA on 5-HT_2_ receptor activity measured using a Gq dissociation BRET-based assay: (h) 5-HT_2A_, (i) 5-HT_2B_, and (j) 5-HT_2C_. 5-HT was used as positive control, and data are represented as mean ± SEM from three to four independent cell culture preparations (*n* = 3–4).

Interactions with monoaminergic systems are a key feature of MDMA.^6,42^ We showed that the novel analogues SDMA and SDA are more potent than MDA at inhibiting SERT and more potent than MDA and MDMA at inhibiting DAT and NET (Figure 1B–D; Table 1). Increased potency was also observed in release assays, where SDA and SDMA induced substrate efflux at lower concentrations than their parent analogues (Figure 1E–G; Table 1). Because of the potency increase, the therapeutic window may be shifted to lower concentrations/doses. Molecular docking studies predicted the fitting of all substances into the binding pocket of the respective transporters and showed an increased fitness score for SDMA and SDA compared to their respective analogues, fitting well with the increased potency observed in the experimental data (Figure 2). To determine how well the compounds can mimic the endogenous substrate, we compared the best scoring poses for each compound with those of the respective endogenous substrates (5-HT for SERT, DA for DAT, and NE for NET), as shown in Figures 2 and 3. Using the transporter as a reference, Figure 2 shows the binding poses of MDA, MDMA, SDA, and SDMA, overlaid with the respective endogenous ligand as observed in the cyro-EM structures. The key structural features important for substrate–transporter interactions are (i) the amine functional group of the substrate that interacts with the conserved aspartate residue present in the substrate binding pocket (D98 in SERT, D79 in DAT, and D75 in NET), (ii) the interaction of the hydrogen bond donor/acceptor function of the hydroxyl group of 5-HT, DA, and NE with the polar residues in the distal end of subpocket B of the transporter, and (iii) the aromatic interactions between the substrate and the substrate binding pocket. The bound ligands show comparable scores and, as expected, higher scores for the ligands with bigger atom counts (Figure 2). The binding poses for all ligands are comparable across the three transporters, which are also consistent with the experimentally observed endogenous ligands: the key features are overlapping (highlighted by dashed circles) or slightly shifted compared to the endogenous ligand. As detailed in Figure 3, the positively charged nitrogen is in a position that allows for interactions with the conserved aspartate residue. The aromatic ring system is interacting with the aromatic side and aliphatic chains of the S1, also if shifted relative to the endogenous ligand. The polar interactions by the hydroxyl group(s) of 5-HT, DA, and NE are slightly different, because MDA, MDMA, SDA, and SDMA are only hydrogen bond acceptors and lack a hydrogen donor functionality. Similar to the NET bound with NE,^43^ the binding poses suggest that these interactions could potentially be formed through water bridges.

**Table 1:** IC_50_ Values Obtained from Uptake Inhibition Assays and EC_50_ and *E*_max_ Values Obtained from Efflux Assays for MDA, MDMA, SDA, and SDMA Represented in Mean ± SD or Mean [95%-CI] EC_50_, pEC_50_, and *E*_max_ values obtained from Gq dissociation BRET-based assays investigating the effects of MDA, MDMA, SDA, and SDMA 5-HT_2_ receptor activities are given in mean or mean ± SEM. 5-HT was used as a positive control. pEC_50_ is defined as the negative logarithm of the EC_50_. (5-HT_2A_: EC_50_ = 6.93 nM; pEC_50_ = 8.16 ± 0.04. 5-HT_2B_: EC_50_ = 2.86 nM; pEC_50_ = 8.54 ± 0.07. 5-HT_2C_: EC_50_ = 1.21 nM; pEC_50_ = 8.92 ± 0.18)

parameter		MDA	MDMA	SDA	SDMA
IC_50_± SD (μM)	DAT/SERT ratio^a^	2.03	0.42	2.42	2.15
	[^3^H]5-HT uptake SERT	22.85 ± 2.27	6.47 ± 0.93	3.09 ± 0.68	4.00 ± 0.87
	[^3^H]DA uptake DAT	11.26 ± 1.28	15.39 ± 2.08	1.28 ± 0.21	1.86 ± 0.58
	[^3^H]MPP^+^uptake NET	3.33 ± 0.47	3.17 ± 0.72	0.85 ± 0.24	0.77 ± 0.39
	[^3^H]MPP^+^uptake OCT1	6.93 ± 2.63	5.09 ± 3.14	3.65 ± 0.27	4.39 ± 2.84
	[^3^H]MPP^+^uptake OCT2	5.13 ± 1.18	5.30 ± 0.25	5.70 ± 0.60	4.13 ± 0.60
EC_50_[95%-CI] (μM)	DAT/SERT ratio	0.65	0.21	0.66	0.37
	SERT mediated [^3^H]5-HT efflux	4.35 [1.47–10.27]	1.95 [1.27–3.01]	0.27 [0.13–0.48]	0.18 [0.046–0.41]
	DAT mediated [^3^H]MPP^+^release	6.72 [2.18–20.56]	9.30 [3.31–22.47]	0.41 [0.23–0.69]	0.49 [0.19–1.22]
	NET mediated [^3^H]MPP^+^release	0.35 [0.13–0.67]	0.73 [0.35–1.34]	0.15 [0.035–0.34]	0.36 [0.22–0.59]
*E*_max_[95%-CI] (%)	SERT mediated [^3^H]5-HT efflux	45.46 [42–48.98]	39.29 [35.53–43.17]	70.5 [64.66–76.72]	71.48 [61.36–83.36]
	DAT mediated [^3^H]MPP^+^ release	67.2 [62.81–71.71]	43.13 [38.7–47.77]	55.37 [48.37–64.43]	35.4 [29.54–43.6]
	NET mediated [^3^H]MPP^+^ release	126.5 [115.3–137.9]	111.1 [105.2–117]	99.61 [92.83–106.7]	103.5 [97.23–109.9]
EC_50_ (nM)	5-HT_2A_	905	3128	340	1396
	5-HT_2B_	139	531	41.5	357
	5-HT_2C_	89	664	48.1	656
pEC_50_ ± SEM	5-HT_2A_	6.04 ± 0.06	5.51 ± 0.07	6.52 ± 0.06	5.86 ± 0.06
	5-HT_2B_	6.86 ± 0.13	6.28 ± 0.21	7.38 ± 0.13	6.45 ± 0.13
	5-HT_2C_	7.05 ± 0.17	6.18 ± 0.17	7.32 ± 0.15	6.18 ± 0.16
*E*_max_ ± SEM (%)	%5-HT response 5-HT_2A_	87.8 ± 2.4	66.0 ± 2.3	92.4 ± 2.0	68.2 ± 1.8
	%5-HT response 5-HT_2B_	103.9 ± 4.9	59.9 ± 5.1	88.2 ± 3.7	70 ± 3.4
	%5-HT response 5-HT_2C_	103.9 ± 6.2	85 ± 6.0	92.9 ± 4.9	79 ± 5.3

a $\mathrm{DAT}/{\mathrm{SERT}}_{\mathrm{ratio}}=\frac{1/\mathrm{DAT}}{1/\mathrm{SERT}}$ .

**Figure 2:**
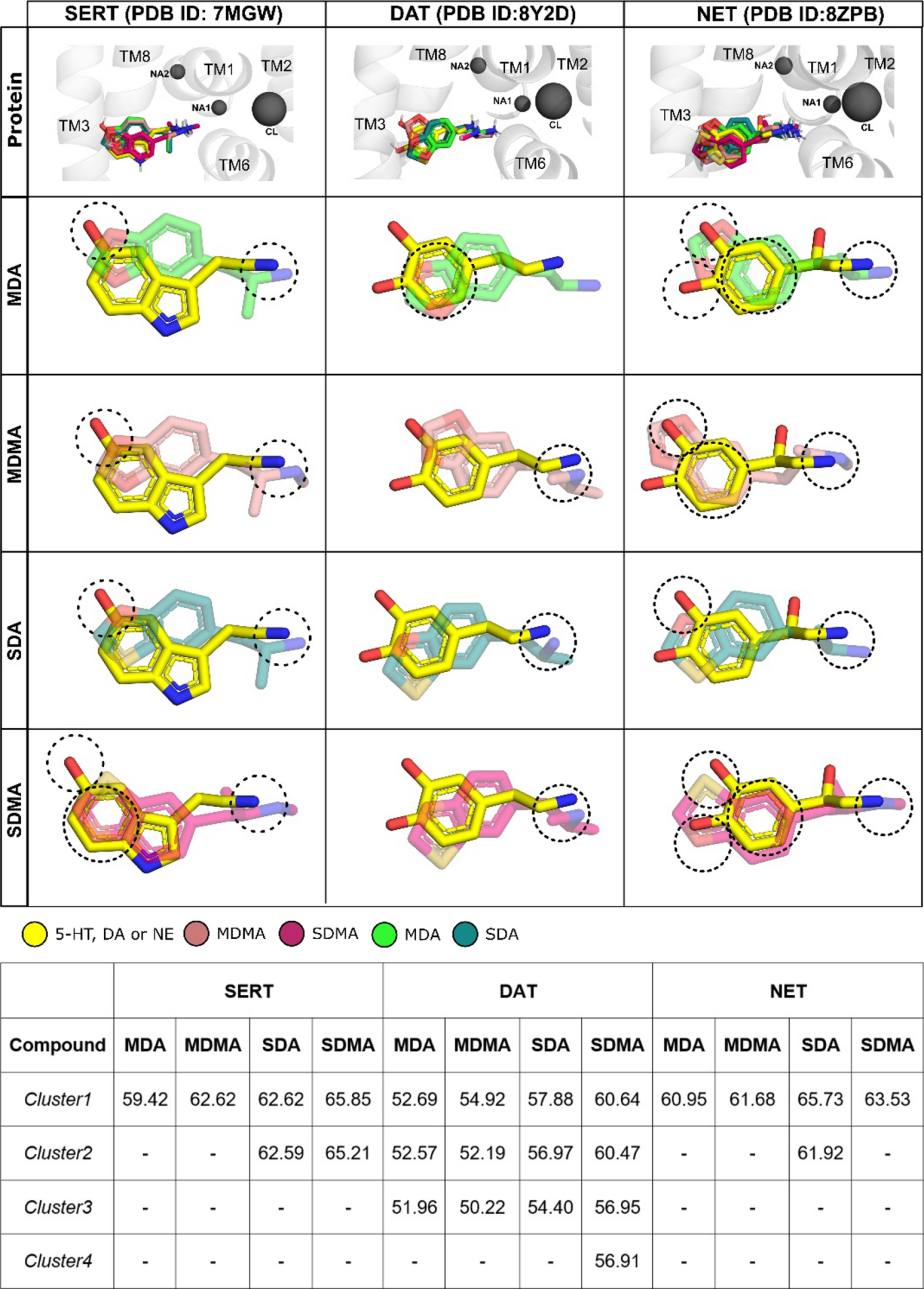
Top-1 docking poses of MDA, MDMA, SDA, and SDMA in SERT, DAT, and NET proteins. The first row depicts the superposition of all compounds’ top-1 binding poses with the cognate substrate in each protein’s orthosteric binding pocket (S1). In the following rows, the compounds are shown individually in superposition with their cognates. The dashed circles represent functional groups of the cognate that are mimicked in the compound structure. Hydrogen atoms are not displayed. Fitness score values for the best pose in each cluster are presented in the table underneath.

**Figure 3:**
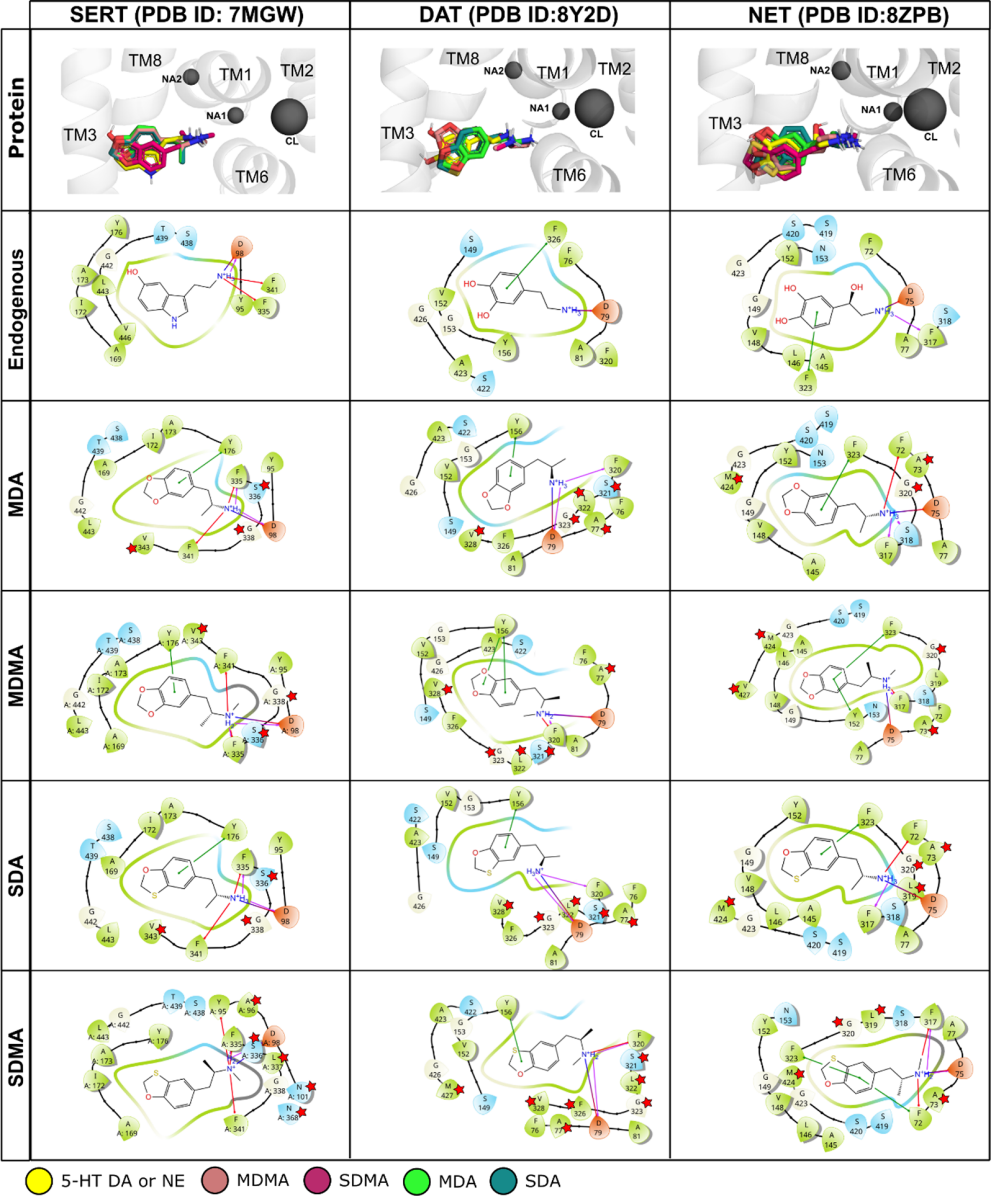
Top-1 docking poses for MDA, MDMA, SDA, and SDMA in SERT, DAT, and NET proteins. The first row represents the superposition of all compounds’ top-1 binding poses with the cognate substrate in each protein’s orthosteric binding pocket (S1). The 2D interaction scheme for each cognate and compound, obtained using Maestro version 13.6.122, is shown in the rows below with the main interactions with the respective protein highlighted. Asterisks indicate residues that do not appear in interaction with the cognate.

Similar to MDMA and MDA, SDMA and SDA act as partial releasers at SERT and DAT and as full releasers at NET. Interestingly, in molecular docking studies, all tested substances closely matched the binding pose of the endogenous substrate NE, placing similar functional groups (the amino group, aromatic ring, and hydroxyl groups) into the respective subpockets. Binding poses in DAT and SERT matched to a lesser degree, showing a degree of correlation with the structural overlay observed in docking and the ability to evoke release. The ability to evoke 5-HT efflux is strongly linked to the therapeutic effects of MDMA,^44,45^ as the prosocial effects of MDMA are lost when its ability to evoke 5-HT release is impaired.^46,47^

There have also been several studies showing that not only SERT, DAT, and NET but also OCTs are important for maintaining monoaminergic homeostasis by sequestering endogenous substrates, which may also be affected by psychostimulant action.^48^ MDMA has been described to inhibit OCT1 and OCT2 in the micromolar range but showed no to little activity at OCT3.^22^ MDA, MDMA, SDA, and SDMA showed a very similar interaction profile, resulting in nearly identical IC_50_ at both transporters (Supplemental Figure 14A–B). Further, we studied the cytotoxicity of the compounds in cells expressing the transporter of interest or in differentiated PC12 cells. There were no significant cytotoxic effects observed, except in PC12 cells, where all compounds exerted cytotoxicity when tested at high micromolar to millimolar concentrations (Supplemental Figure 20). These high concentrations are not physiologically relevant, as dosing in clinical trials results in peak concentrations that usually do not exceed the low micromolar range.^18,19,49^

Neurotoxic effects of MDMA in particular have also been partially attributed to its metabolites in some studies, such as catechol and quinone metabolites due to the production of free radicals.^28–31^ In this study, the main metabolic pathways for both MDMA analogues *in vitro* were demethylenation, *N*-demethylation for SDMA (SDMA-M1), and *N*-acetylation for SDA (SDA-M6). These metabolites showed the highest intensities across all metabolites (Table 2; Figure 4A–B). Comparing the results against the *in vitro* metabolism of MDMA, all metabolic reactions of MDMA were also found for the two analogues including *N*-demethylation and demethylenation followed by methylation or sulfation^50–52^ (Table 2; Supplemental Figure 17). In addition, hydroxylation, *N*-oxygenation, and *N*-acetylation could be identified as potential routes of metabolism in this study. In summary, demethylenation and hydroxylation are suitable analytical targets for both compounds in toxicological urine screenings, as they exhibited a high intensity *in vitro* and allowed the differentiation of the two analogues. *In vitro* metabolic stability screening was conducted to assess the susceptibility of a compound to *in vivo* biotransformation.^53^ The depletion of the compounds during incubation with pHLM was employed to ascertain their metabolic stability, which was expressed as *t*_1/2_, CL_int, micr_, and CL_int_ extrapolated to whole liver dimensions. CL_int_ is defined as the maximum biotransformative activity of the liver toward a drug in the absence of other physiological determinants such as hepatic blood flow and drug binding to constituents within the blood mixture.^53^ To preclude the possibility of nonspecific protein binding, protein concentrations should be minimized and the concentration of the compound during incubation should be maintained below the Michaelis–Menten concentration (*K*_m_). As no information on *K*_m_ values was available for the tested compounds, a low compound concentration was used in the assay as recommended earlier.^53^ Nonmetabolic degradation of the substances can be excluded based on control incubations without pHLM and subsequent *t*-test, which showed no significant differences in the natural logarithms of the peak area between 0 min and control incubations. MDA exhibited only weak metabolic degradation, as neither half-life nor clearance values could be determined (>150 min). This observation also extended to MDMA.^22^ SDA and SDMA can be classified as low clearance compounds^54^ with half-lives of 61 and 71 min, respectively (Figure 4C–E). The prolonged *in vitro* half-lives of MDMA and MDA compared to SDA and SDMA could be explained by the fact that both MDMA and MDA are substrates of CYP2D6, leading to autoinhibition.^36,55^

**Table 2:** Detection of SDA and SDMA and Their Phase I and II Metabolites in Pooled Human Liver Microsomes and/or S9 Fraction and Reported Phase I and/or Phase II Metabolites of MDA and MDMA in Literature in Pooled Human Liver Microsomes and/or S9 ^50,52,56,57^ Together with Their Metabolite Identification Numbers (ID), Calculated Exact Mass of the Protonated Molecule (M + H^+^), Elemental Composition, and Retention Time (RT)

metabolite-ID	metabolic reaction	calculated exact mass, *m*/*z*	elemental composition	RT, min^b^
MDA		180.1019	C_10_H_13_NO_2_	N.A.
MDA-M1^a^	demethylenation	168.1019	C_9_H_13_NO_2_	N.A.
MDA-M2^a^	demethylenyl-methylation	182.1175	C_10_H_15_NO_2_	N.A.
MDA-M3^a^	demethylenyl-methylation	182.1175	C_10_H_15_NO_2_	N.A.
MDMA		194.1176	C_11_H_15_NO_2_	N.A.
MDMA-M1^a^	*N*-demethylation	180.1019	C_10_H_13_NO_2_	N.A.
MDMA-M2^a^	demethylenation	182.1176	C_10_H_15_NO_2_	N.A.
MDMA-M3^a^	demethylenyl-methylation	196.1332	C_11_H_17_NO_2_	N.A.
MDMA-M4^a^	demethylenyl-methylation	196.1332	C_11_H_17_NO_2_	N.A.
MDMA-M5^a^	demethylenyl-sulfation	262.0744	C_10_H_15_NO_5_S	N.A.
MDMA-M6^a^	demethylenyl-sulfation	262.0744	C_10_H_15_NO_5_S	N.A.
MDMA-M7^a^	demethylenyl-methyl-sulfation	274.0755	C_11_H_17_NO_5_S	N.A.
SDA		196.0791	C_10_H_14_ONS	5.3
SDA-M1	hydroxylation	212.0740	C_10_H_14_O_2_NS	4.2
SDA-M2	*N*-oxygenation	212.0740	C_10_H_14_O_2_NS	5.8
SDA-M3	demethylenation	184.0791	C_9_H_14_ONS	4.0
SDA-M4	demethylenyl-methylation	198.0947	C_10_H_14_ONS	4.6
SDA-M5	demethylenyl-methylation	198.0947	C_10_H_14_ONS	5.0
SDA-M6	*N*-acetylation	238.0896	C_12_H_16_O_2_NS	7.6
SDMA		210.0947	C_11_H_16_ONS	5.5
SDMA-M1	*N*-demethylation	196.0791	C_10_H_14_ONS	5.3
SDMA-M2	hydroxylation	226.0896	C_11_H_16_O_2_NS	4.4
SDMA-M3	*N*-oxygenation	226.0896	C_11_H_16_O_2_NS	6.1
SDMA-M4	demethylenation	198.0947	C_10_H_14_ONS	4.3
SDMA-M5	demethylenyl-methylation	212.1104	C_11_H_18_ONS	4.7
SDMA-M6	demethylenyl-methylation	212.1104	C_11_H_18_ONS	5.1
SDMA-M7	demethylenyl-sulfation	278.0515	C_10_H_16_O_4_NS_2_	4.2
SDMA-M8	*N*-demethyl-*N*-acetylation	238.0896	C_12_H_16_O_2_NS	7.6

a Literature data.

b N.A., no retention time available for the used analytical method.

**Figure 4:**
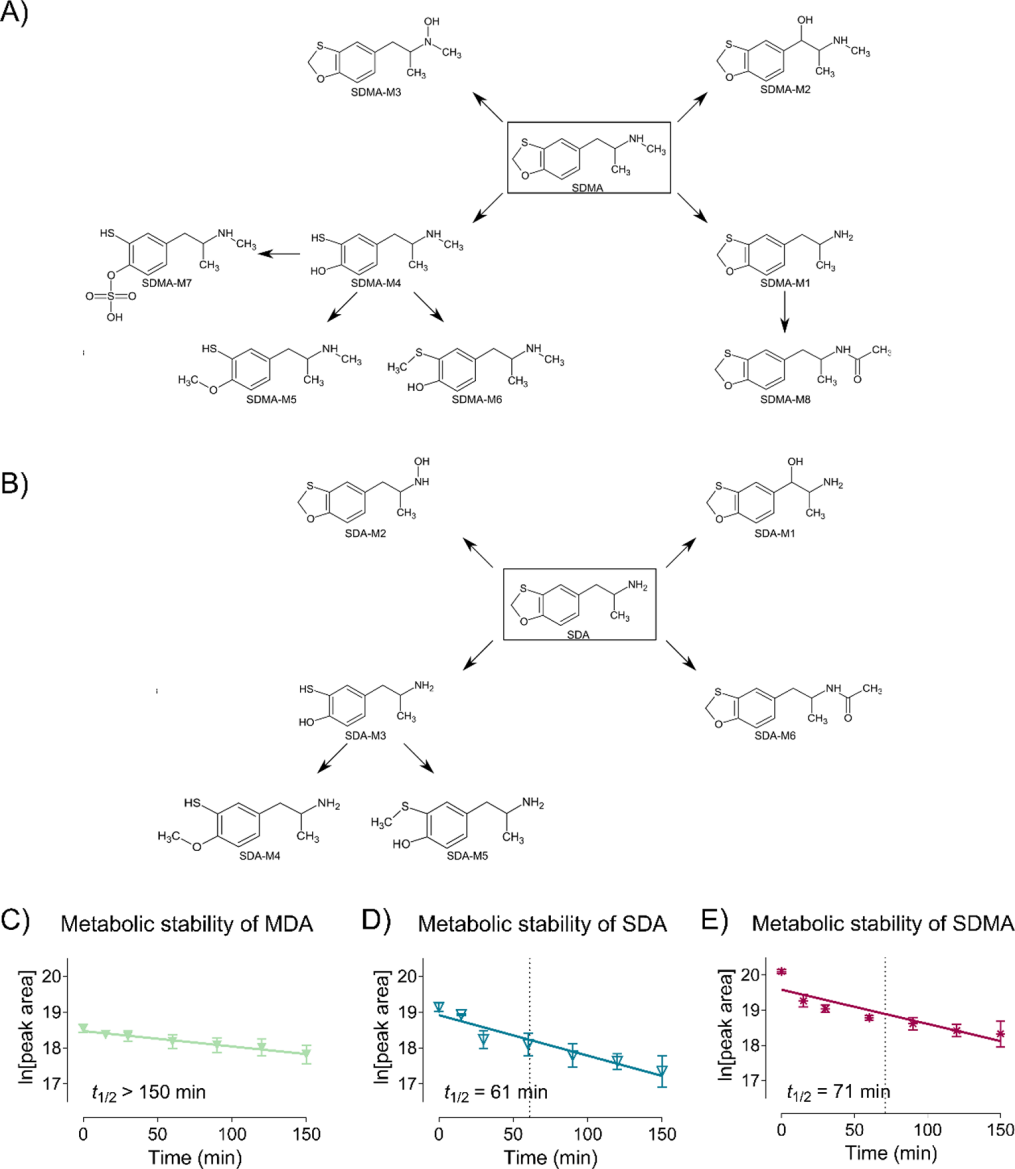
Metabolic pathways of (A) SDMA and (B) SDA in incubations with pooled human liver microsomes and/or the S9 fraction. Metabolite-IDs correspond to Table 2. Metabolic stability of (C) MDA, (D) SDA, and (E) SDMA in incubations with pooled human liver microsomes (pHLM). Data of MDMA was published previously.^22^ Incubation time is plotted versus the natural logarithm of the peak area of the compound. Points indicate mean values (*n* = 2), *t*_1/2_ = in vitro half-life. M = Metabolite-ID.

Since MDMA is known to interact with members of the 5-HT_2_ receptor family and to elicit some of its psychoactive effects through these interactions, we explored the ability of the novel analogues to activate these receptor subtypes.^11,12,58^ In particular, 5-HT_2A_ agonism is commonly linked with psychedelic effects and can predict psychedelic potential based on Gq dissociation efficacy.^59^ This receptor is further associated with the serotonin syndrome, a rare but severe adverse event that can occur after administration of serotonergic drugs, which can lead to excessive extracellular 5-HT concentrations.^60^ We observed that MDA and SDA showed increased potency and efficacy at 5-HT_2A_ compared to MDMA and SDMA (Figure 1H; Supplemental Figure 13; Table 1). Additionally, SDA showed significantly increased and prolonged core temperature changes compared with MDMA and SDMA (Figure 5J–K), which could potentially be related to its increased 5-HT_2A_ receptor activation. Both SDA and SDMA induced hyperlocomotion (Figure 5A, D), with a ceiling effect observed at the 25 mg/kg dose. This is a high dose that can induce other behaviors, such as stereotypies, which may alter locomotor activity. In fact, this is reflected in the time-course data (Supplemental Figure 22), where an initial peak in hyperlocomotion is followed by a sudden decrease, especially for SDMA. Moreover, the efficacy of the compounds at inducing locomotion was studied at 10 mg/kg, revealing a significantly higher efficacy for SDA in comparison to both SDMA and MDMA (Figure 5G), which could also potentially augment the temperature increase and may be causally linked to the 5-HT_2A_ receptor activation. To assess whether the test compounds can activate the 5-HT_2A_ receptor *in vivo*, we evaluated their effects on the head-twitch response (HTR) in male C57BL/6J mice, a rodent behavioral proxy for human psychedelic effects^61^ that is mediated by activation of 5-HT_2A_-Gq signaling.^59^ Neither MDMA nor SDMA induced an appreciable increase in HTR counts over the baseline levels. By contrast, both MDA and SDA showed evidence of an orderly dose-related increase in HTR counts peaking at 3 mg/kg, although the magnitude of the response induced by SDA was not large enough to achieve statistical significance in posthoc pairwise comparisons (Supplemental Figure 23A–C).^62^ We recently discovered that the magnitude of the HTR (the maximum number of HTR counts induced by any dose of a 5-HT_2A_ agonist) in mice is related to 5-HT_2A_-Gq efficacy and agonists with *E*_max_ <70% relative to 5-HT in BRET assays do not induce the HTR at all.^59^ Because the 5-HT_2A_-Gq *E*_max_ of MDA and SDA is >70% and the 5-HT_2A_-Gq *E*_max_ of MDMA and SDMA is <70%, it is notable that only MDA and SDA showed evidence of activity in the HTR assay. The intrinsic efficacy of MDMA and SDMA to activate 5-HT_2A_-Gq signaling may not be high enough to induce head twitches, indicating that those compounds are unlikely to produce psychedelic effects in humans. Indeed, in clinical trials, MDMA does not mimic most of the characteristic psychedelic effects produced by LSD and psilocybin.^2,63^ Furthermore, while pretreatment with the 5-HT_2A_ antagonist ketanserin blocks virtually all of the effects of psychedelics such as LSD and psilocybin in humans,^64,65^ pretreatment with ketanserin has relatively little effect on the response to MDMA,^66^ which seems to be largely mediated by increases in 5-HT efflux via SERT.^46^ The absence of a high level of 5-HT_2A_ activation may be advantageous for MDMA and its analogues in the context of their therapeutic use, as strong psychedelic effects may not always be desirable in clinical settings. Although different doses were used for HTR testing in comparison to the other *in vivo* experiments (the doses tested in HTR were based on the salt form of the compounds instead of the free base), the tested ranges were comparable overall.

**Figure 5:**
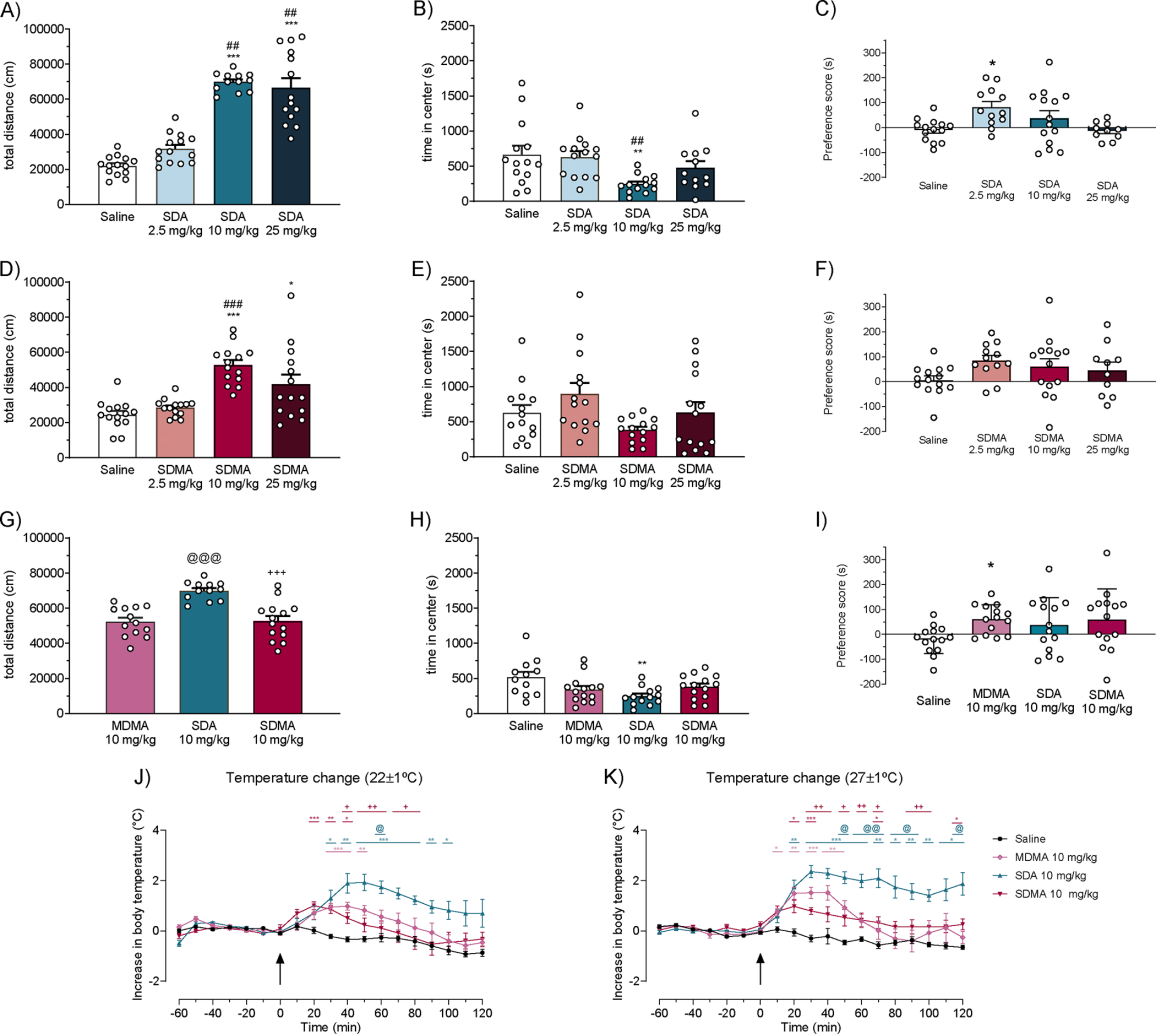
Effects of increasing doses of SDA (A) and SDMA (D) and comparative effects of MDMA, SDA, and SDMA at 10 mg/kg (G) on cumulative horizontal locomotor activity in mice. Data is presented as mean ± SD of the total distance (cm) traveled in 120 min and was analyzed with Dunn’s multiple-comparisons test: * *p* < 0.05 and *** *p* < 0.001 vs saline, ## *p* < 0.01 and ### *p* < 0.001 vs 2.5 mg/kg, @@@ *p* < 0.001 vs MDMA, and +++ *p* < 0.001 vs SDA. *N* = 12–14 per group. (B, E, H) Open field test (thigmotaxis) in male Swiss CD-1 mice. Bars represent mean ± SD of time in the center (s). Dunn’s/Tukey’s multiple-comparisons test: **p < 0.01 vs saline and ## *p* < 0.01 vs 2.5 mg/kg. *N* = 12–14/group. (C, F, I) Conditioned place preference test in mice. Data is presented as mean ± SD of the preference score (difference between the time spent in the drug-paired compartment on the test day and the preconditioning day). *N* = 10–14 per group. Dunn’s multiple-comparisons test: *p < 0.05 vs saline. (J, K) Core body temperature changes in mice at 22 ± 1 °C (J) and 28 ± 1 °C (K). Data is normalized to the baseline temperature of each respective animal, presented as mean ± SD of the increase in core body temperature for each time point and analyzed using Tukey’s multiple comparisons test (*n* = 5–7 per group) | * *p* < 0.05 vs saline, ** *p* < 0.01 vs saline, *** *p* < 0.001 vs saline, @ *p* < 0.05 vs MDMA, @@ *p* < 0.01 vs MDMA, + *p* < 0.05 vs SDA, ++ *p* < 0.01 vs SDA. For panels H–I, the saline group is composed of pooled saline controls from panels B, E and C, F, respectively. Doses were calculated based on the free base form of each compound.

Thigmotaxis was evaluated to assess potential anxiety-like effects induced by the compounds. When comparing the tested doses of SDA and SDMA (2.5, 10, and 25 mg/kg) with saline (Figure 5B, E, respectively), a significant decrease in thigmotaxis, reflected by reduced time spent in the center of the arena, was observed only for SDA at 10 mg/kg. To further explore this effect, we compared the 10 mg/kg doses of SDA and SDMA with MDMA and saline (Figure 5H). Consistently, only SDA significantly decreased the time spent in the center of the arena, indicating anxiety-like inducing effects of the substance. 5-HT_2B_ receptor agonism is associated with cardiotoxic effects of serotonergic substances.^67,68^ We show that our substances exhibit similar interactions compared with their respective analogue (Figure 1I; Supplemental Figure 13; Table 1). Combined with the increased potency at monoamine transporters and a subsequent possible reduction of therapeutic dose, the off-target effects mediated by 5-HT_2A_ and 5-HT_2B_ activation could therefore be potentially minimized.

In addition, while MDMA has been described to induce rewarding effects,^69^ which was confirmed in our studies, neither SDA nor SDMA induced significant rewarding effects at 10 mg/kg compared to saline (Figure 5I), pointing to a slightly reduced reward potential, which may be offset by their agonist activity at 5-HT_2C_ receptors (Figure 1J; Supplemental Figure 13; Table 1).^70^ It must be pointed out that so often, an all-or-nothing effect is observed in the CPP paradigm with a threshold above which significant preference is obtained. Thus, the drug’s rewarding potency obtained through the CPP paradigm can be best determined from the threshold dose when comparing different drugs.^71^ To more thoroughly characterize the rewarding effects of these compounds, additional doses were tested. Notably, SDA showed a significant preference score at the lowest dose tested (Figure 5C). Although SDMA also resulted in a positive preference score at the same dose, it did not reach significance (Figure 5F). Although no significant rewarding effects were observed for SDMA in the CPP paradigm, further studies using complementary behavioral models to examine other MDMA-like addictive properties, such as reinforcing, extinction, relapse, and sensitization, are needed to fully assess its abuse liability and overall safety profile.

In conclusion, we show that SDMA could prove to be a viable candidate for replacing MDMA as it shows a similar pharmacological profile with increased potency in the SLC6 transporter family, possibly shifting the therapeutic window. Both SDA and SDMA show clearance rates faster than those of MDA and MDMA, which therefore might help prevent the prolonged induction of cytotoxic effects. Likely reduced reward potential also offers promising insights, suggesting a lower risk for dependence. However, SDA showed some disadvantages regarding 5-HT_2A_ and 5-HT_2B_ interaction, hyperthermia, and thigmotaxis compared to MDMA and SDMA. Similar interactions of SDMA and MDMA with 5-HT_2_ receptors and thermoregulation at the same dose and lack of HTR indicate that a dose reduction could also reduce side effects. Overall, these findings highlight SDMA as a promising alternative to MDMA, offering potential therapeutic advantages with a favorable pharmacological and toxicological profile while paving the way for further research to explore its full potential.

## Materials and Methods

### Drugs and Reagents

MDMA hydrochloride (HCl; MW = 229.7 g/mol; Cat #13971) and MDA HCl (MW = 215.7 g/mol; Cat #11554) were obtained from Cayman Chemical (Tallinn, Estonia). SDA and SDMA were synthesized by Mihkal GmBH (Allschwil, Switzerland) as oxalate salts through the chemical synthesis pathway shown in Supplemental Figure 1 and described in detail in the Supplemental File (Supplemental Figures 2–11). Further used material is also listed in the Supplemental File. MDA, MDMA, SDA, and SDMA were all tested as racemic mixtures.

### Cell Culture

Stable mono- or polyclonal human embryonic kidney (HEK293) cell lines were used as described prior.^72^ HEK293 cells were authenticated in April 2024 by CLS Cell Lines Dienstleistung GmbH in Germany. Detailed information can be found in the Supporting Information.

### Uptake Inhibition Assay

Uptake inhibition assays were performed as previously described.^72^ Cells were seeded at a density of 36,000 cells per well into poly-d-lysine (PDL) coated 96-well plates 1 day prior to the experiment. At the beginning, cells were washed once with 200 μL of Krebs-HEPES-buffer (KHB) consisting of 120 mM NaCl, 3 mM KCl, 2 mM CaCl_2_·2H_2_O, 2 mM MgCl_2_·6H_2_O, and 20 mM d-glucose. The pH was adjusted to 7.3 with NaOH. Substances of interest were dissolved in DMSO or H_2_O (Milli-Q) at a concentration of 100 mM. Cells were preincubated with 50 μL/well of prediluted substance of interest (SOI; diluted in KHB) in the respective concentration for 5 min (SERT, DAT, NET) or 10 min (OCT1, OCT2). Subsequently, the solution was replaced with 50 μL/well uptake solution containing the diluted substance and tritiated substrate (SERT: 0.1 μM [^3^H]­5-HT; DAT: 0.1 μM [^3^H]­DA; NET: 0.02 μM [^3^H]­MPP^+^; OCT1, OCT2:0.05 μM [^3^H]­MPP^+^) for 1 min (SERT, DAT) and 3 min (NET, OCT1, OCT1). Uptake was stopped by washing the cells with 200 μL of KHB. To quantify beta emission with liquid scintillation counting (Wallac 1450 MicroBeta TriLux Liquid Scintillation Counter & Lumi; GMI, Ramsey, MN, USA), 200 μL of Ultima Gold XR scintillation cocktail (Cat #6013119; Revvity; Waltham, MA, USA) was added to each well. Total uptake (100%) was defined as the uptake in the absence of SOI, and nonspecific uptake (0%) was defined as uptake in the presence of an established inhibitor (SERT: 3 μM paroxetine; DAT, NET: 50 μM GBR12909; OCT1, OCT2:100 μM decynium-22) and subtracted from each value. Half-maximal inhibitory concentrations (IC_50_) were calculated from fitting a sigmoidal concentration–response ((fixed hill slope = –1) ( $Y=\mathrm{Bottom}+\frac{\mathrm{Top}-\mathrm{Bottom}}{1+10^{(\left(\mathrm{LogIC}50-X\right)}}$ −) in GraphPad Prism 10.3.1 (GraphPad Software Inc., San Diego, CA, USA).

### Release Assay

Release assays were performed modified from previously published protocols.^73^ HEK293 cells expressing the transporter of interest were seeded into 6 channel flow channel ibidi slides with a density of 80,000 cells/channel a day prior to the experiment. On the day of the experiment, the cells were preloaded with 50 μL/well 0.05 μM [^3^H]­MPP^+^ (DAT), 0.015 μM [^3^H]­MPP^+^ (NET), or 0.05 μM [^3^H]­5-HT (SERT) for 20 min at 37 °C. The slides were then transferred to the release apparatus,^73^ where they were continuously perfused with a flow rate of 0.5 mL/min. Cells were washed for 12–15 min with constant KHB superfusion to reach a basal efflux rate. Afterward, fractions were collected every 2 min. The first three fractions were cells superfused with KHB to establish basal release. The subsequent five fractions were conducted with the SOI in the indicated concentration. At the end of the release experiment, the remaining cells were lysed by superfusion with sodium dodecyl sulfate solution (SDS; 1%) to retrieve the remaining radioactivity within the cells. All fractions were collected in 8 mL vials containing 2 mL of scintillation cocktail for subsequent counting. The measured amount of tritiated substrate in the respective fraction is expressed as the percentage of tritiated substrate at the start of the respective fraction. The percentage of efflux was calculated by subtracting the mean baseline efflux from the mean efflux at the plateau and normalized to the efflux of a known full releaser (Supplemental Figure 12). Half maximal effective concentration (EC_50_) was calculated from fitting a sigmoidal concentration–response (fixed hill slope = 1) ( $Y=\mathrm{Bottom}+\frac{\mathrm{Top}-\mathrm{Bottom}}{1+10^{(\left(\mathrm{LogEC}50-X\right)}}$ ) in GraphPad Prism 10.3.1 (GraphPad Software Inc., San Diego, CA, USA).

### Serotonin 5-HT_2A/2B/2C_ Receptor Activity - Gq Dissociation Bioluminescence Resonance Energy Transfer (BRET) Assays

The 5-HT_2_ activities of MDA, MDMA, SDA, and SDMA were determined using a Gq dissociation BRET assay as previously described.^22,74,75^ In brief, HEK293T cells (ATCC; RRID:CVCL_0063) in DMEM containing 10% dialyzed FBS (dFBS) were transfected with the human 5-HT_2_ receptor, Gαq-Rluc8, β3, and GFP2-γ9 DNA constructs in a 1:1:1:1 ratio for 5-HT_2A_ and 5-HT_2B_ receptors and a 1:2:2:2 ratio for 5-HT_2C_ assays. The next day, cells were seeded in poly l-lysine-coated 96-well white assay plates at a density of approximately 40,000 cells/well in DMEM containing 1% dFBS. The following day, medium was replaced with 60 μL/well of drug buffer (1× HBSS, 20 mM HEPES, pH 7.4) and equilibrated for at least 15 min at 37 °C in a humidified incubator. Compounds were diluted in drug buffer containing 0.3% fatty-acid free BSA and 0.03% ascorbic acid and added to the respective wells (30 μL/well). After 45 min of incubation at 37 °C in a humidified incubator, 10 μL/well coelenterazine 400a (5 μM final concentration; Nanolight Technology) was added. Plates were incubated for another 15 min at 37 °C in a humidified incubator before measurement with a PheraStarFSX or ClarioStar Plus (BMG Labtech; Cary, NC). BRET ratios of 510 nm/400 nm luminescence were calculated and normalized to % positive control (5-HT) stimulation. A concentration response curve was fitted using nonlinear regression. All experiments were performed with at least three independent cell culture preparations and in duplicate. Half maximal effective concentration (EC_50_) was calculated from fitting a sigmoidal concentration–response (fixed hill slope = 1) ( $Y=\mathrm{Bottom}+\frac{\mathrm{Top}-\mathrm{Bottom}}{1+10^{(\left(\mathrm{LogEC}50-X\right)}}$ ) in GraphPad Prism 10.3.1 (GraphPad Software Inc., San Diego, CA, USA). pEC_50_ and *E*_max_ values were analyzed in GraphPad Prism 10.3.1 by using one-way ANOVA followed by Tukey’s post hoc test (Supplemental Tables 1–6).

### Cytotoxicity Assay

Cytotoxicity assays were performed according to the manufacturer protocol (G4000, Promega, WI, USA) for HEK cells and described previously^76^ for PC12 cells (detailed information in the Supporting Information).

### Confocal Microscopy

The membrane expression of the expressed transporters was evaluated by confocal microscopy as described elsewhere^77^ and is described in detail in the Supporting Information.

### Molecular Docking

We used the cryo-EM structures of SERT (PDB ID: 7MGW),^78^ DAT (PDB ID: 8Y2D),^79^ and NET (PDB ID: 8ZPB)^43^ bound to their respective cognate ligands 5-HT, DA, and NE. Prior to docking calculation, we removed all the nonprotein atoms (except for the cotransported sodium ions NA_1_, NA_2_, and chloride ion Cl) and added hydrogen atoms to the structures using PyMol version 2.5.0.^80^ The ligands were built using YASARA version 21.12.19 ^81^ as an (*R*)-enantiomer, with protonation for pH 7, whereby the primary amino group carries a positive charge. Next, we performed energy minimization using YASARA to obtain the optimized geometries of the ligands. The calculations were carried out in vacuum using the AMBER 96 force field^82^ with an 0.8 nm force cutoff and particle mesh Ewald algorithm to treat long-range electrostatic interactions. After removing the structural stress by energy minimization, annealing simulation was performed using a time step of 2 fs with atom velocities scaled down by 0.9 every 10th step until energy converged to less than 0.05 kJ/mol per atom during 200 steps. The same protocol was applied to the cognate substrate extracted from the cryo-EM structure.

The ligands were docked into the transporters with the cotransported ions bound using the GOLD (Genetic Optimization for Ligand Docking) software version 2022.2.0.^83^ For each system, ten docking solutions were generated. Calculations were performed by using a cavity formed by a 0.8 nm radius sphere placed on the center of mass of the respective endogenous substrates (5-HT for SERT, DA for DAT, and NE for NET). The calculation was performed with a rigid receptor. The docking poses were ranked using the GoldScore^84^ scoring function (“fitness score”). The number of Genetic Algorithm (GA) runs was set to 10, and all other parameters were set as the default. The selection of the GoldScore scoring function was based on redocking calculations (Supplemental Figure 15), where SERT, DAT, and NET were docked with their respective endogenous substrates, following the protocol described in the Supporting Information.

### Hepatic Metabolism

LC-HRMS/MS conditions used for metabolism studies are described in detail in the Supporting Information.

#### Pooled Human Liver Microsome Incubation for Identification of *In Vitro* Phase I Metabolites

Incubation using pooled human liver microsomes (pHLM) was prepared according to published procedures.^85,86^ SDMA or SDA was dissolved freshly in methanol and subsequently diluted with 0.1 M phosphate buffer to obtain the required concentrations. Incubations were performed using a final concentration of 0 or 25 μM of the respective compound and 1 mg of protein/mL pHLM at 37 °C. The final incubation mixtures also contained 90 mM phosphate buffer, 5 mM isocitrate, 5 mM Mg^2+^, 1.2 mM NADP^+^, 200 U/mL SOD, and 0.5 U/mL isocitrate dehydrogenase. A final incubation volume of 50 μL was obtained. The reaction was stopped after 60 min by adding 50 μL of ice-cold acetonitrile containing L-tryptophan-d_5_ (25 μM) and trimipramine-d_3_ (2.5 μM). Samples were centrifuged for 2 min at 18,407*g*. To exclude interfering and nonmetabolically formed compounds, additional incubations were performed without the substrate (blank) and without enzymes (negative control). For each group, two replicates were prepared.

#### Pooled Human Liver S9 Fraction Incubation for Identification of *In Vitro* Phase I and II Metabolites

Pooled human liver S9 fraction incubation was performed in accordance with minor deviations from a previous publication.^50^ Incubations using the pooled human liver S9 fraction (pHLS9) at a final protein concentration of 2 mg/mL were performed after 10 min of preincubation at 37 °C with 50 μg/mL alamethicin (UGT reaction mixture solution B), 90 mM phosphate buffer (pH 7.4), 2.5 mM Mg^2+^, 2.5 mM isocitrate, 0.6 mM NADP^+^, 0.8 U/mL isocitrate dehydrogenase, 101 U/mL SOD, 0.1 mM AcCoA, 2.3 mM acetyl carnitine, and 8 U/mL acetylcarnitine transferase. Afterward, 2.5 mM UDP-glucuronic acid (UGT reaction mixture solution A), 40 μM PAPS, 1.2 mM SAM, 1 mM DTT, and 10 mM GSH were added.

Reactions were started by adding 25 μM SDMA or SDA. Mixtures (final volume 150 μL; *n* = 2 each) were incubated for a maximum of 360 min. After 60 min, a 60 μL sample was transferred to a new reaction tube containing 20 μL of ice-cold acetonitrile containing L-tryptophan-d_5_ (25 μM) and trimipramine-d_3_ (2.5 μM) to stop the reaction. The remaining mixtures were incubated for an additional 300 min and thereafter stopped with 30 μL of ice-cold acetonitrile containing L-tryptophan-d_5_ (25 μM) and trimipramine-d_3_ (2.5 μM). Afterward, tubes were cooled for 30 min at −20 °C and centrifuged at 18,407*g* for 2 min, and supernatants were transferred to autosampler vials and analyzed by LC-HRMS/MS. Blank incubation (without substrate) and control incubation (without pHLS9) were done to confirm the absence of interfering compounds and to identify not metabolically formed compounds. All incubations were performed in duplicate. The amount of organic solvent was below 1%.^87^

#### Metabolic Stability Studies

*In vitro* metabolic stability was estimated by using substrate depletion of SDMA, SDA, and MDA according to Wagmann et al.^88^ Incubations were performed with pHLM in accordance with the pHLM incubation with the following variations: 0.5 μM substrate concentrations were used, and incubations were stopped after 0, 15, 30, 60, 90, 120, and 150 min by addition of 50 μL of ice-cold acetonitrile containing L-tryptophan-d5 (25 μM) and trimipramine-d_3_ (2.5 μM) as internal standards. Additionally, control incubations (*n* = 2) without pHLM were prepared to assess enzyme independent degradation of parent compounds and stopped after 150 min. All incubations were performed in duplicate. Mixtures were centrifuged (18,407*g* for 2 min), and the resulting supernatants were transferred into LC vials and analyzed by LC-HRMS/MS. The degradation of parent compounds was further assessed by calculating the natural algorithm of the absolute peak area of the analyte in HR full scan. Statistical evaluation was performed using GraphPad Prism 10.00 (GraphPad Software, San Diego, CA, USA). A *t*-test was conducted to determine if there were significant differences between ln­[peak area]_initial_ values and ln­[peak area] values in control incubations without pHLM using the following settings: unpaired; two-tailed; significance level, 0.05; confidence intervals, 99%.

Calculations were performed according to the equations of Baranczewski et al.:^53^

$$\ln{\left[\mathrm{peak}\;\mathrm{area}\right]}_{\mathrm{remaining}}=\ln{\left[\mathrm{peak}\;\mathrm{area}\right]}_{\mathrm{initial}}-k\cdot t\to t_{1/2}\left(\min\right)=\frac{\ln\left(2\right)}{k}$$ 1

$${\mathrm{CL}}_{\mathrm{int},\mathrm{micr}}=\frac{\ln\left(2\right)}{t_{1/2}\left(\min\right)}\cdot \frac{{\left[V\right]}_{\mathrm{incubation}}\left(\mathrm{mL}\right)}{{\left[P\right]}_{\mathrm{incubation}}\left(\mathrm{mg}\right)}$$ 2

$${\mathrm{CL}}_{\mathrm{int}}={\mathrm{CL}}_{\mathrm{int},\mathrm{micr}}\left(\frac{\mathrm{mL}}{\min\cdot \mathrm{mg})}\right)\cdot \frac{\left[\mathrm{Liver}](g\right)}{\left[\mathrm{BW}](\mathrm{kg}\right)}\cdot \mathrm{SF}\left(\frac{\mathrm{mg}}{g}\right)$$ 3

where *k* is the slope of the linear regression fit, *t*_1/2_ is the *in vitro* half-life, CL_int, micr_ is the microsomal intrinsic clearance, CL_int_ is the intrinsic clearance, [V]_incubation_ is the incubation volume, which is 0.05, [P]_incubation_ is the microsomal protein amount in incubation, which is 0.05, $\frac{\left[\mathrm{Liver}\right]}{\left[\mathrm{BW}\right]}$ is the liver weight normalized by body weight,^89^ which is 26, and SF is the scaling factor microsomal protein per gram of liver, which is 33.^53^

### *In Vivo* Behavioral Experiments

Subjects and housing conditions are described in the Supporting Information. Animals were subjected to a light–dark cycle and had free access to food and water (standard laboratory diet, Panlab SL, Barcelona, Spain). All animal care and experimental protocols were approved by the Animal Ethics Committee of the University of Barcelona under the supervision of the Autonomous Government of Catalonia. These protocols complied with the guidelines of the European Community Council (2010/63/EU), as amended by Regulation (EU) 2019/1010, and adhered to the ARRIVE guidelines^90^ for reporting animal experiments. In alignment with the 3R principles, we minimized the number of animals used by testing only three out of the four compounds (MDMA, SDA, and SMDA), as MDA would only serve as an additional control. This approach reduces animal use (Replacement) while maintaining experimental relevance, avoids unnecessary duplication (Reduction), and ensures adherence to ethical standards in animal research (Refinement). The mice were randomly assigned to experimental groups, and efforts were made to minimize both animal use and suffering. The sample size for all behavioral experiments was determined using GPower software.

#### Subjects

Male Swiss CD-1 mice (Janvier, Le Genest, France), weighing 30–35 g and aged 6–8 weeks, were used for HLA, thigmotaxis, CPP, and core body temperature experiments. Animals were housed under temperature-controlled conditions (22 ± 1 °C) with a 12 h light/dark cycle and had free access to food and water (standard laboratory diet, Panlab SL, Barcelona, Spain). Doses for HLA, thigmotaxis, CPP, and core body temperature experiments were calculated based on the free base form of each compound. For HTR experiments, male C57BL/6J mice (6–8 weeks old) were obtained from Jackson Laboratories (Bar Harbor, ME, USA) and housed up to four per cage with a reversed light-cycle (lights on at 1900 h, off at 0700 h). Food and water were provided *ad libitum*, except during behavioral testing. Testing was conducted between 1000 and 1830 h. Doses for HTR experiments were calculated based on the respective salt forms of the compounds.

#### Horizontal Locomotor Activity (HLA) and Thigmotaxis

A habituation phase was performed to reduce the stress and novelty associated with handling and injection. During this phase, which lasted for two consecutive days, mice received an intraperitoneal (ip) saline injection and were immediately placed into a black Plexiglas Open Field (OF) arena (25 × 25 × 40 cm) under low-light conditions and white noise for 30 min. On the test day, their horizontal locomotor activity (HLA) was measured. In short, mice received an i.p. injection of saline (5 mL/kg) or a dose of 2.5, 10, or 25 mg/kg (calculated as MDMA free base) of SDA and SDMA or a 10 mg/kg dose of MDMA and were immediately placed in the OF arena with the same light and noise conditions. HLA was video monitored for 2 h, and a specific tracking software (Smart 3.0 Panlab, Barcelona, Spain) was used to measure their total traveled distance (in cm). Center Versus Periphery: The thigmotactic effects of the test compounds, widely considered as anxiety-like effects, were assessed through an OF test. Each mouse was individually placed in the center of an open-field arena (25 cm length × 25 cm width × 40 cm height). The time spent, in seconds, in the center (8 × 8 cm) or the periphery of the arena was monitored for 2 h (Smart 3.0 Panlab, Barcelona, Spain). Data was further analyzed in GraphPad Prism 10.3.1 by Kruskal–Wallis followed by Dunn’s test for total HLA and one-way ANOVA followed by Tukey’s multiple comparisons test for OF and two-way ANOVA followed by Tukey’s multiple comparisons test for time-profile analysis (Supplemental Tables 15–23).

#### Conditioned Place Preference (CPP)

For this experiment, an apparatus with two distinct compartments, differing in tactile and visual cues, connected by a central corridor, was used. The conditioned place preference (CPP) procedure consisted of three phases: preconditioning, conditioning, and postconditioning. In the preconditioning phase (day 0), mice were placed in the middle of the corridor and allowed to freely explore both compartments for 15 min. The time spent in each compartment was recorded and monitored using specific tracking software (Smart 3.0 Panlab, Barcelona, Spain). In the conditioning phase (day 1–day 8), access to the corridor was closed. Mice received an i.p. injection of the corresponding drug (MDMA, SDA, or SDMA) on conditioning days 1, 3, 5, and 7 and were immediately placed into one of the compartments for 30 min. On alternate days,^2,4,6,8^ mice received a saline i.p. injection and were placed in the other compartment for 30 min. Mice in the control group received a saline injection in every session. The compartments and sessions in which the drug was administered were randomized. In the postconditioning phase (test day), the same conditions as the preconditioning phase were reinstated. A preference score was determined by calculating the difference in time spent in the drug-paired compartment between the post- and preconditioning phases. Data was further analyzed in GraphPad Prism 10.3.1 by one-way ANOVA or Kruskal–Wallis test followed by Dunn’s multiple-comparisons test (Supplemental Tables 24–26).

#### Core Body Temperature

Changes in body temperature were measured by means of subcutaneously implanted temperature transponders (IPTT-300; Bio Medic Data Systems, Inc., DE, USA). Seven mice were used per group. Four days before the experiment, mice were anesthetized using isoflurane, and the transponders were subcutaneously placed in the interscapular zone with a transponder injector. 10% iodine antiseptic solution was applied to the injection points to prevent infections. Mice were returned to their home cages and checked regularly until complete cicatrization. On the day of the experiment, mice were allowed to habituate for 1 h. Subsequently, core body temperature measures were taken for 1 h, every 10 min before administration, using a DAS-8007 Wireless Reader System (Bio Medic Data Systems, Inc.), to establish a baseline. Afterward, mice received i.p. injections of saline, MDMA, SDA, or SDMA (10 mg/kg i.p. calculated as MDMA free base), and temperature was assessed every 10 min for 2 h. The experiment was performed at 22 ± 1 and 27 ± 1 °C with naïve mice. Data was further analyzed in GraphPad Prism 10.3.1 with the mixed-effects model followed by Tukey’s multiple comparisons test (Supplemental Tables 27–28).

#### Head-Twitch Response (HTR)

The HTR was assessed using a head-mounted magnet and a magnetometer detection coil.^91^ Mice were anesthetized, a small incision was made in the scalp, and a small neodymium magnet was attached to the dorsal surface of the cranium by using dental cement. Following a two week recovery period, HTR experiments were carried out in a well-lit room with at least 7 days between sessions to avoid carryover effects. Mice (*N* = 4–7/group) received intraperitoneal (IP) injections of vehicle (saline) or test drug (5 mL/kg injection volume), and then activity was recorded in a glass cylinder surrounded by a magnetometer coil for 30 min. Doses for HTR experiments were calculated based on the respective salt forms of the compounds. Coil voltage was low-pass filtered (1 kHz cutoff frequency), amplified, digitized (20 kHz sampling rate, 16-bit ADC resolution), and saved to disk using a Powerlab 8/35 data acquisition system with LabChart software (ver. 8.1 ADInstruments, Colorado Springs, CO, USA). To detect head twitches, events in the recordings were transformed to scalograms, deep features were extracted using the deep convolutional neural network ResNet-50, and then the images were classified using a support vector machine (SVM).^61^ Head twitch counts were analyzed using one-way ANOVA. Posthoc comparisons were made using Dunnett’s test. Significance was demonstrated by surpassing an α-level of 0.05 (Supplemental Tables 29–31).

## Supplementary Material



## Data Availability

The data that supports the findings of this study are available from the corresponding author, H.H.S., upon reasonable request.

## Notes

Additional information on the synthesis process of novel compounds and experimental details of cell culture, cell viability, confocal microscopy, and hepatic metabolism. Additional results for superfusion assays, 5-HT receptor activation, OCT uptake inhibition, molecular docking, hepatic metabolism, cytotoxicity assays, transporter membrane expression, and *in vivo* behavioral assays including statistical values (PDF)

## Abbreviations

MDMA: 3,4-Methylenedioxymethamphetamine
MDA: 3,4-Methylenedioxyamphetamine
SDA: 1-(1,3-Benzoxathiol-5-yl)­propan-2-amine
SDMA: 1-(1,3-Benzoxathiol-5-yl)-*N*-methylpropan-2-amine
SERT: Serotonin transporter
DAT: Dopamine transporter
NET: Norepinephrine transporter
5-HT: 5-Hydroxytryptamine (serotonin)
DA: Dopamine
NE: Norepinephrine (noradrenaline)
IC_50_: Half-maximal inhibitory concentration (concentration needed to inhibit uptake by 50%)
EC_50_: Half-maximal effective concentration (concentration needed to achieve 50% of the maximum effect)
