# Correction to “Next-Generation MDMA Analogue SDMA: Pharmacological and Metabolic Insights”

**DOI:** 10.1021/acschemneuro.6c00136

**Published:** 2026-03-04

**Authors:** Nina Kastner, Núria Nadal-Gratacós, Selina Hemmer, Leticia Alves da Silva, John L. McKee, Tamara Hell, Giulia Cicalese, Marion Holy, Fatemeh Kooti, Kathrin Jäntsch, Ricarda Baron, Naomi Shacham, Bruna Cuccurazzu, Adam L. Halberstadt, John D. McCorvy, Thomas Stockner, Markus R. Meyer, Raúl López-Arnau, Matthias Grill, Harald H. Sitte

Correction 1: Table 1. The analyses were conducted using the correct data, and all results, interpretations, and conclusions in the paper remain fully accurate. The issue is limited to the presentation of values in the table.

**Table 1:** IC_50_ Values Obtained from Uptake Inhibition Assays and EC_50_ and *E*_max_ Values Obtained from Efflux Assays for MDA, MDMA, SDA, and SDMA Represented in Mean ± SD or Mean [95%-CI][Table-fn tbl1-fn0] ^a^

Compound		MDA	MDMA	SDA	SDMA
**IC**_**50**_ **± SD (μM)**	**DAT/SERT ratio** ^a^	2.03	0.42	2.42	2.15
	**[** ^**3**^ **H]5-HT uptake SERT**	22.85 ± 2.27	6.47 ± 0.93	3.09 ± 0.68	4.00 ± 0.87
	**[** ^**3**^ **H]DA uptake DAT**	11.26 ± 1.28	15.39 ± 2.08	1.28 ± 0.21	1.86 ± 0.58
	**[** ^**3**^ **H]MPP** ^**+**^ **uptake NET**	3.33 ± 0.47	3.17 ± 0.72	0.85 ± 0.24	0.77 ± 0.39
	**[** ^**3**^ **H]MPP** ^**+**^ **uptake OCT1**	6.93 ± 2.63	5.09 ± 3.14	3.65 ± 0.27	4.39 ± 2.84
	**[** ^**3**^ **H]MPP** ^**+**^ **uptake OCT2**	5.13 ± 1.18	5.30 ± 0.25	5.70 ± 0.60	4.13 ± 0.60
**EC**_**50**_ **[95%-CI] (μM)**	**DAT/SERT ratio** ^a^	0.65	0.21	0.66	0.37
	**SERT mediated [** ^**3**^ **H]5-HT efflux**	4.35 [1.47–10.27]	1.95 [1.27–3.01]	0.27 [0.13–0.48]	0.18 [0.046–0.41]
	**DAT mediated [** ^**3**^ **H]MPP** ^**+**^ **release**	6.72 [2.18–20.56]	9.30 [3.31–22.47]	0.41 [0.23–0.69]	0.49 [0.19–1.22]
	**NET mediated [** ^**3**^ **H]MPP** ^**+**^ **release**	0.35 [0.13–0.67]	0.73 [0.35–1.34]	0.15 [0.035–0.34]	0.36 [0.22–0.59]
**E**_**max**_ **[95%-CI] (%)**	**SERT mediated [** ^**3**^ **H]5-HT efflux**	71.48 [61.36–83.36]	70.5 [64.66–76.72]	45.46 [42–48.98]	39.29 [35.53–43.17]
	**DAT mediated [** ^**3**^ **H]MPP** ^**+**^ **release**	35.4 [29.54–43.6]	55.37 [48.37–64.43]	67.2 [62.81–71.71]	43.13 [38.7–47.77]
	**NET mediated [** ^**3**^ **H]MPP** ^**+**^ **release**	103.5 [97.23–109.9]	99.61 [92.83–106.7]	126.5 [115.3–137.9]	111.1 [105.2–117]
**EC**_**50**_ **(nM)**	**5-HT**_**2A**_	905	3128	305	1396
	**5-HT**_**2B**_	139	531	41.5	357
	**5-HT**_**2C**_	89	348	48.1	656
**pEC**_**50**_ **± SEM**	**5-HT**_**2A**_	6.04 ± 0.06	5.51 ± 0.07	6.52 ± 0.06	5.86 ± 0.06
	**5-HT**_**2B**_	6.86 ± 0.13	6.28 ± 0.21	7.38 ± 0.13	6.45 ± 0.13
	**5-HT**_**2C**_	7.05 ± 0.17	6.45 ± 0.19	7.32 ± 0.15	6.18 ± 0.16
**E**_**max**_ **± SEM (%)**	**%5-HT response 5-HT**_**2A**_	87.8 ± 2.4	66. 0 ± 2.3	92.4 ± 2.0	68.2 ± 1.8
	**%5-HT response 5-HT**_**2B**_	103.9 ± 4.9	59.9 ± 5.1	88.2 ± 3.7	70 ± 3.4
	**%5-HT response 5-HT**_**2C**_	89.3 ± 5.5	79.7 ± 5.8	92.9 ± 4.9	79 ± 5.3

a EC_50_, pEC_50_ and *E*_max_ values obtained from Gq dissociation BRET-based assays investigating effects of MDA, MDMA, SDA, and SDMA 5-HT_2_ receptor activity are given in mean or mean ± SEM. 5-HT was used as positive control. pEC_50_ is defined as the negative logarithm of the EC_50_. (5-HT_2A_: EC_50_ = 6.93 nM; pEC_50_ = 8.16 ± 0.04. 5-HT_2B_: EC_50_ = 2.86 nM; pEC_50_ = 8.54 ± 0.07. 5-HT_2C_: EC_50_ = 1.21 nM; pEC_50_ = 8.92 ± 0.18).

b ${DAT/SERT}_{ratio}=\frac{1/DAT}{1/SERT}$

Correction 2: p 4733, second paragraph, 1^st^ sentence.

Thigmotaxis was evaluated to assess potential anxiety-like effects induced by the compounds. When comparing the tested doses of SDA and SDMA (2.5, 10, and 25 mg/kg) with saline (Figure 5B and 5E, respectively), a significant increase in thigmotaxis, reflected by reduced time spent in the center of the arena, was observed only for SDA at 10 mg/kg.

