# Supplementary material for: Next-Generation MDMA Analogue SDMA: Pharmacological and Metabolic Insights
Source: ACS Chem Neurosci. 2025 Dec 2;16(24):4725–40. doi: 10.1021/acschemneuro.5c00782 (PMC12715756; doi:10.1021/acschemneuro.5c00782)
Supplement: Supplementary file 1 [file cn5c00782_si_001.pdf]

Supplemental Information

Title:

Next-Generation MDMA Analogue SDMA: Pharmacological and Metabolic Insights

Authors:

Nina Kastner[1], Núria Nadal-Gratacós[2], Selina Hemmer[3], Leticia Alves da Silva[1], John L. McKee[4], Tamara Hell[5], Giulia Cicalese[2], Marion Holy[1], Fatemeh Kooti[1], Kathrin Jäntsch[1], Ricarda Baron[1], Naomi Shacham[6], Bruna Cuccurazzu[6], Adam L. Halberstadt[6], John D. McCorvy[4,7], Thomas Stockner[1], Markus R. Meyer[3], Raúl López-Arnau[2], Matthias Grill[5], Harald H. Sitte[1,6,7,*]

Affiliations / Author Information:

(1) Institute of Pharmacology, Center for Physiology and Pharmacology, Medical University of Vienna, 1090 Vienna, Austria

(2) Department of Pharmacology, Toxicology and Therapeutic Chemistry, Faculty of Pharmacy, Pharmacology Section and Institute of Biomedicine (IBUB), University of Barcelona, 08028 Barcelona, Spain

(3) Experimental and Clinical Toxicology and Pharmacology, Center for Molecular Signaling (PZMS), PharmaScienceHub (PSH), Saarland University, 66424 Homburg, Germany

(4) Department of Cell Biology, Neurobiology and Anatomy, Medical College of Wisconsin, Milwaukee, Wisconsin, USA

(5) MiHKAL GmbH, 6362 Stansstad, Switzerland

(6) Department of Psychiatry, University of California San Diego, 9500 Gilman Drive, La Jolla, California, USA

(7) Department of Pharmacology and Toxicology, Neuroscience Research Center, Medical College of Wisconsin, Milwaukee, WI 53226, USA

(8) Hourani Center for Applied Scientific Research, Al-Ahliyya Amman University, 19328 Amman, Jordan

(9) AddRess Centre for Addiction Research and Science, Medical University of Vienna, 1090 Vienna, Austria

Corresponding author:

* Harald H. Sitte

harald.sitte@meduniwien.ac.at

+43-1-40160-31323

Waehringerstrasse 13A, 1090 Vienna

Table of Contents

1 Supplementary Material and Methods 3

1.1 Drugs and Reagents 3

1.2 Synthesis of SDMA 3

1.2.1 Preparation of Benzo[d][1,3]oxathiole 4

1.2.2 Preparation of Benzo[d][1,3]oxathiole-5-carbaldehyde 5

1.2.3 Preparation of 5-(2-nitroprop-1-en-1-yl)benzo[d][1,3]oxathiole 5

1.2.4 Preparation of 1-(benzo[d][1,3]oxathiol-5-yl)propan-2-amine (SDA) 6

1.2.5 Preparation of ethyl (1-(benzo[d][1,3]oxathiol-5-yl)propan-2-yl)carbamate 6

1.2.6 Preparation of 1-(benzo[d][1,3]oxathiol-5-yl)-N-methylpropan-2-amine (SDMA) 7

1.2.7 Purity and Characterization 8

1.3 Cell Culture 10

1.4 Cell viability (PC12; WST-8 assay) 10

1.5 Confocal Microscopy 10

1.6 Hepatic Metabolism: LC-HRMS/MS conditions 11

2 Supplementary Results 12

2.1 Superfusion Release Assays: Control Substances 12

2.2 5-HT receptors statistical analysis 13

2.3 OCT Uptake Inhibition 15

2.4 Molecular docking 16

2.5 Hepatic Metabolism 17

2.6 Cytotoxicity assays in differentiated PC12 cells and SERT, DAT and NET expressing HEK cells 23

2.7 Transporter membrane expression 25

2.8 *In vivo* behavioral assays 28

3 References 34

1 Supplementary Material and Methods

1.1 Drugs and Reagents

Vanoxerine (GBR12909; Cat #D052), monensin (Cat #M5273) were purchased from Sigma-Aldrich (St. Louis, MO, United States), and paroxetine HCl (Cat #AB 439408) was obtained from abcr GmbH (Karlsruhe, Germany). Dulbecco's Modified Eagle Medium (DMEM) high glucose (4.5 g/L) with L-glutamine (Cat #DMEM-HA), fetal bovine serum (FBS; Cat #FBS-11A) and geneticin (G-418 sulfate solution; 50 mg/mL; Cat #G418-B) were purchased from Capricorn Scientific GmbH (Ebsdorfergrund, Germany). 1-Ethyl-2-((1-ethylquinolin-2(1H)ylidene)methyl)quinolinium iodid (Decynium22) was purchased from Chemos (Cat #ST00067). Radioactively labelled substrates [^{3}H]5-HT (1 mCi; Cat #NET498) and [^{3}H]1-methyl-4-phenylpyridinium ([^{3}H]MPP$^+$; 250 µCi; Cat #NET914) were obtained from Revvity (Waltham, MA, USA). Sodium chloride (NaCl), potassium chloride (KCl), calcium dichloride dihydrat ($CaCl_2 \cdot 2H_2O$), magnesium dichloride hexahydrate ($MgCl_2 \cdot 6H_2O$), 20 mM and 4-(2-hydroxyethyl)-1-piperazineethanesulfonic acid (HEPES) were obtained from Merck (Darmstadt, Germany) or Thermo Fisher Scientific (Waltham, MA, USA)

Acetonitrile (LC-MS grade), ammonium acetate, ammonium formate, formic acid (LC-MS grade), methanol (LC-MS grade), and nicotinamide adenine dinucleotide phosphate (NADP$^+$) were obtained from VWR (Darmstadt, Germany). Acetylcarnitine, acetylcarnitine transferase, acetyl coenzyme A (AcCoA), dipotassium hydrogenphosphate (K_2HPO_4), potassium dihydrogenphosphate (KH_2PO_4) were obtained from Sigma Aldrich (Taufkirchen, Germany). S-(5'-adenosyl)-L-methionine (SAM), dithiothreitol (DTT), isocitrate, isocitrate dehydrogenase, magnesium chloride ($MgCl_2$), 3'-phosphoadenosine-5'phosphosulfate (PAPS), reduced glutathione (GSH), superoxide dismutase (SOD), and trimipramine-d_3 (internal standard, IS) were from Merck (Taufkirchen, Germany). L-Tryptophan-d_5 was purchased from Alsachim (Illkirch-Graffenstaden, France).
Pooled human liver microsomes (pHLM, 20 mg protein/mL, 330 pmol total CYP/mg protein, 35 donors), pHLS9 (20 mg microsomal protein/mL, 30 individual donors), uridine 5'-diphospho-glucuronosyltransferase (UGT) reaction mixture solution A (25 mM UDP-glucuronic acid), and UGT reaction mixture solution B (250 mM Tris HCl, 40 mM $MgCl_2$, and 125 µg/mL alamethicin) were obtained from Corning (Amsterdam, Netherlands), After delivery, the enzymes, pHLM, and pHLS9 were thawed at 37°C, aliquoted, snap-frozen in liquid nitrogen, and stored at -80°C until use.

1.2 Synthesis of SDMA

An overview of the SDA and SDMA synthesis pathway is depicted in Supplemental Figure 1. Detailed synthesis description can be found in the following subsections

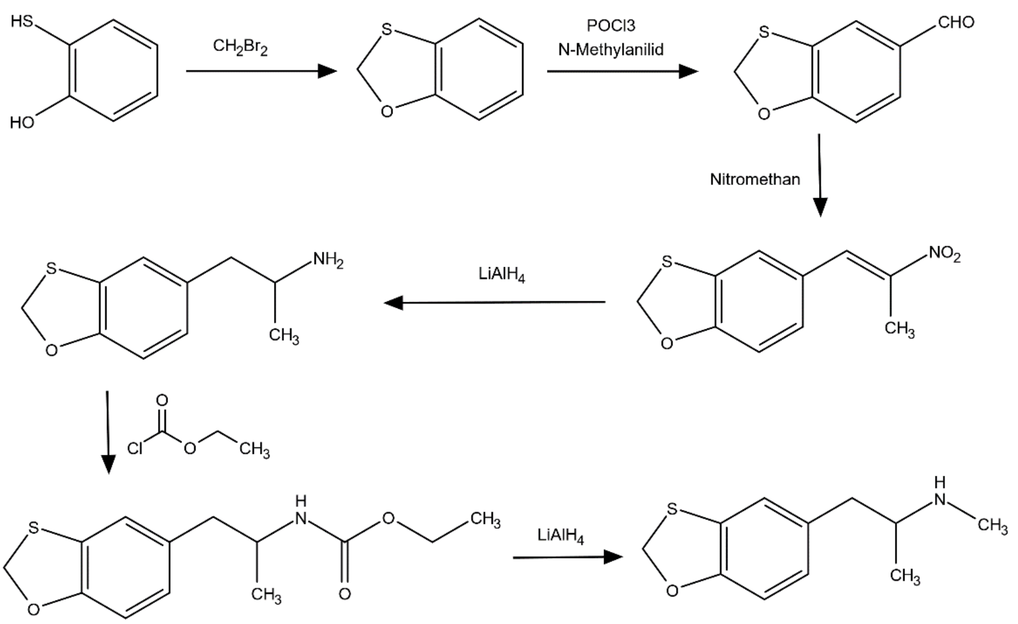

Supplemental Figure 1: Chemical synthesis pathway for SDA and SDMA.

1.2.1 Preparation of Benzo[d][1,3]oxathiole

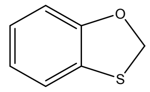

benzo[*d*][1,3]oxathiole
Chemical Formula: C_7H_6OS
Exact Mass: 138.01
Molecular Weight: 138.18

Supplemental Figure 2: Structure of Benzo[d][1,3]oxathiole

A 2 L-reactor vessel was charged with dibromomethane (100 ml, 1.28 mol), deionized water (500 ml), Adogen 464 (approx. 16.6 g) and was stirred under reflux for 1h. Subsequently, a solution of mercaptophenol (80 ml, 0.717 mol) in sodium hydroxide solution (80 g sodium hydroxide in 360 ml deionized water) was added over a period of 90 min. The resulting suspension was stirred under reflux for 2-3 h. According to TLC analytics, after this period all parts of starting material (mercaptophenol) have been consumed. For setting the pH-value acidic, to the crude reaction mixture (pH-value of 14) was added diluted hydrochloric acid dropwise. As a result, the solution was adjusted to approx. pH 1. The reaction mixture was extracted several times with dichloromethane. Subsequently, the combined organic phase was evaporated to yield an oily residue. The crude product was purified via column over silica using the eluent mixture hexane/dichloromethane in a ratio of 8:2. This yielded 85.1 g of product (84 %). TLC (254 nm) [hexane/ethylacetate 9:1]: R_f 0.7

1.2.2 Preparation of Benzo[d][1,3]oxathiole-5-carbaldehyde

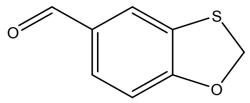

benzo[*d*][1,3]oxathiole-5-carbaldehyde
Chemical Formula: $C_8H_6O_2S$
Exact Mass: 166,01
Molecular Weight: 166,19

Supplemental Figure 3: Structure of Benzo[d][1,3]oxathiole-6-carbaldehyde

A 500 ml round flask was charged with Benzo[d][1,3]oxathiole (59.32 g, 0.43 mol) and N-Methylformanilide (100 ml, 0.81 mol). Over a period of 30 min Phosphoryl chloride (60 ml, 0.645 mol) was added dropwise under stirring at 25°C. Subsequently, the reaction mixture was stirred at 55°C for 72h. For quenching purposes, the reaction mixture was poured on 500 g ice. The resulting emulsion was extracted using methyl-tert-butylether. The combined organic phases were washed with sodium hydrogen carbonate solution as well as sodium chloride solution. The organic phase was dried over magnesium sulfate. After evaporation of the ether, this process yields 43 g of crude product with benzo[d][1,3]oxathiole as main impurity. The crude product was purified via column over silica using the eluent mixture hexane/dichloromethane in a ratio of 8:2. This yielded 8.11 g of product (16.5%). During this purification step, nearly all unreacted starting material can be recycled. As a result, the total yield can be amended to 44%. TLC (254 nm/366 nm) [dichloromethane]: R_f 0.6

1.2.3 Preparation of 5-(2-nitroprop-1-en-1-yl)benzo[d][1,3]oxathiole

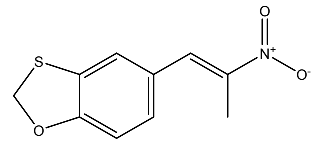

(*E*)-5-(2-nitroprop-1-en-1-yl)benzo[*d*][1,3]oxathiole
Chemical Formula: $C_{10}H_9NO_3S$
Molecular Weight: 223.25

Supplemental Figure 4: Structure of 5-(2-nitroprop-1-en-1-yl)benzo[d][1,3]oxathiole

A 1L two-neck-round flask with mounted Dean-Stark-apparatus was charged with benzo[d][1,3]oxathiole-5-carbaldehyde (20.6 g, 0.121 mol), nitroethane (48.3 ml, 0.675 mol), dimethylamine hydrochloride (21.75g, 0.267 mol) and potassium fluoride (1.20 g, 0.02 mol). The starting materials were mostly dissolved in 400 ml toluene. The reaction mixture was stirred under reflux at 110°C. Water separation inside the Dean-Stark-apparatus can be observed after the first hours. After 2d stirring at 110°C, TLC shows complete consumption of aldehyde-starting material. The reaction mixture was filtered through P4-glass filter to remove all solid salts. Subsequently, the filtrate was purified via silica column using toluene as eluent. This yielded 24.5 g of yellow-orange solid as product (91%). TLC (254 nm/VIS) [toluene]: R_f 0.5 (orange spot)

1.2.4 Preparation of 1-(benzo[d][1,3]oxathiol-5-yl)propan-2-amine (SDA)

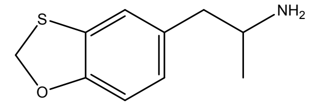

1-(benzo[*d*][1,3]oxathiol-5-yl)propan-2-amine
Chemical Formula: $C_{10}H_{13}NOS$
Exact Mass: 195,07
Molecular Weight: 195,28

Supplemental Figure 5: Structure of 1-(benzo[d][1,3]oxathiol-5-yl)propan-2-amine (SDA)

A 3 L-reactor vessel was charged with 400 ml lithium aluminum hydride solution (1M in tetrahydrofuran) and 200 ml of dry tetrahydrofuran. A solution of 5-(2-nitroprop-1-en-1-yl)benzo[d][1,3]oxathiole (16.07 g , 72 mmol) in 200 ml tetrahydrofuran was added dropwise over a period of 45 min. Subsequently, the reaction mixture was stirred at 70°C for 20h. The reaction mixture was diluted with 300 ml tetrahydrofuran and cooled to 5°C via ice bath. For quenching purposes, sodium sulfate decahydrate (80 g) was added in small portions to the stirred reaction mixture. The resulting slurry was filtered, and the filter cake was eluted with methyl-tert-butylether. The combined filtrates were evaporated to dryness and afforded 7.47 g colorless oil (53%). TLC (254 nm) [dichloromethane/methanol 9:1 + 1% ammonia]: R_f 0.35

1.2.5 Preparation of ethyl (1-(benzo[d][1,3]oxathiol-5-yl)propan-2-yl)carbamate

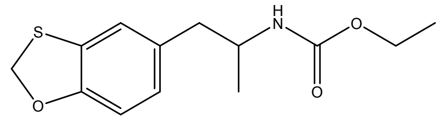

ethyl (1-(benzo[*d*][1,3]oxathiol-5-yl)propan-2-yl)carbamate
Chemical Formula: $C_{13}H_{17}NO_3S$
Exact Mass: 267,09
Molecular Weight: 267,34

Supplemental Figure 6: Structure of ethyl (1-(benzo[d][1,3]oxathiol-5-yl)propan-2-yl)carbamate

SDA (4.88 g, 25 mmol) was dissolved in 100 ml dichloromethane and triethylamine (4.5 ml, 32.5 mmol) was added. The reaction solution was stirred at room temperature. Ethyl chloroformate (2.61 ml, 27.5 mmol) was added dropwise over a period of 15 min. The reaction mixture was stirred for 2h at room temperature. After this period, TLC shows complete conversion to the corresponding carbamate. The reaction mixture was diluted with 100 ml dichloromethane and washed with diluted hydrochloric acid, water and brine. The organic phase was purified over a plug of silica. The combined filtrates were evaporated to yield 4.83 g yellow oil (72%). TLC (254 nm) [dichloromethane/methanol 9:1 + 1% ammonia]: R_f 0.85

1.2.6 Preparation of 1-(benzo[d][1,3]oxathiol-5-yl)-N-methylpropan-2-amine (SDMA)

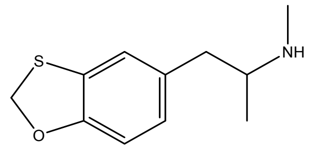

1-(benzo[*d*][1,3]oxathiol-5-yl)-*N*-methylpropan-2-amine
Chemical Formula: $C_{11}H_{15}NOS$
Exact Mass: 209,09
Molecular Weight: 209,31

Supplemental Figure 7: Structure of 1-(benzo[d][1,3]oxathiol-5-yl)-N-methylpropan-2-amine (SDMA)

A 3 L-reactor vessel was charged with 200 ml lithiumaluminium hydride solution (1M in tetrahydrofuran) and 300 ml of dry tetrahydrofuran. A solution of ethyl (1-(benzo[d][1,3]oxathiol-5-yl)propan-2-yl)carbamate (4.83 g, 18 mmol) in 100 ml tetrahydrofuran was added dropwise over a period of 30 min. Subsequently, the reaction mixture was stirred at 70°C for 3h. The reaction mixture was diluted with 300 ml methyl-tert-butylether and cooled to 5°C via ice bath. For quenching purposes, sodium sulfate decahydrate (17 g) was added in small portions to the stirred reaction mixture. The resulting slurry was filtered and the filter cake was eluted with methyl-tert-butylether. The combined filtrates were evaporated to dryness, and the residue was purified via column over silica using the eluent mixture dichloromethane/methanol in a ratio of 9:1 + 1% ammonia. This yielded 1.58 g of product (42 %). TLC (254 nm) [dichloromethane/methanol 9:1 + 1% ammonia]: R_f 0.35

1.2.7 Purity and Characterization

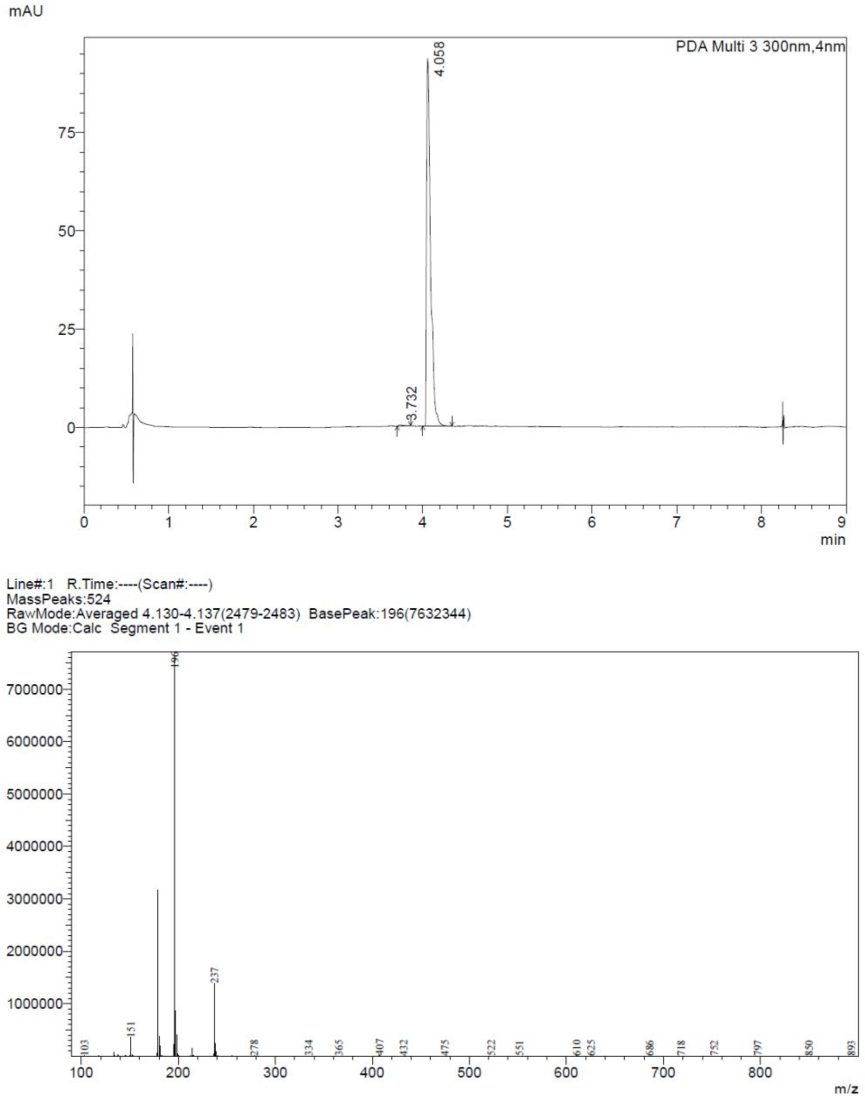

Supplemental Figure 8: LC-MS of SDA (*m/z* 196.0791), purity SDA oxalate = 99.7%

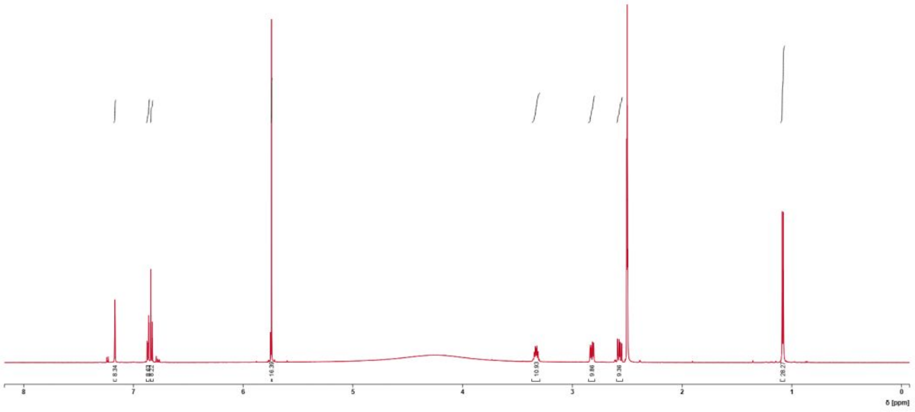

Supplemental Figure 9: ^{1}H-NMR-Spectra (600MHz, d^6-DMSO, 303K) of SDA. δ (ppm) = 7.17 (d, 1H, H_{arom}); 6.88 (d, 1H, H_{arom}); 6.82 (d, 1H, H_{arom}); 5.73 (s, 2H, $H_{methylen}$); 3.35 (m, 1H, $H_{methylen}$); 2.83 (dd, 1H, $H_{methylen}$); 2.56 (dd, 1H, $H_{methylen}$); 1.05 (d, 3H, H_{methyl})

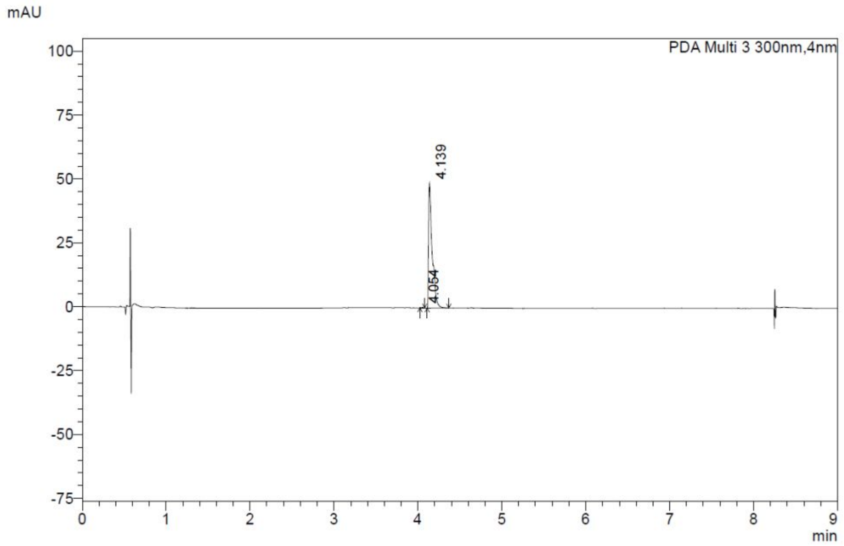

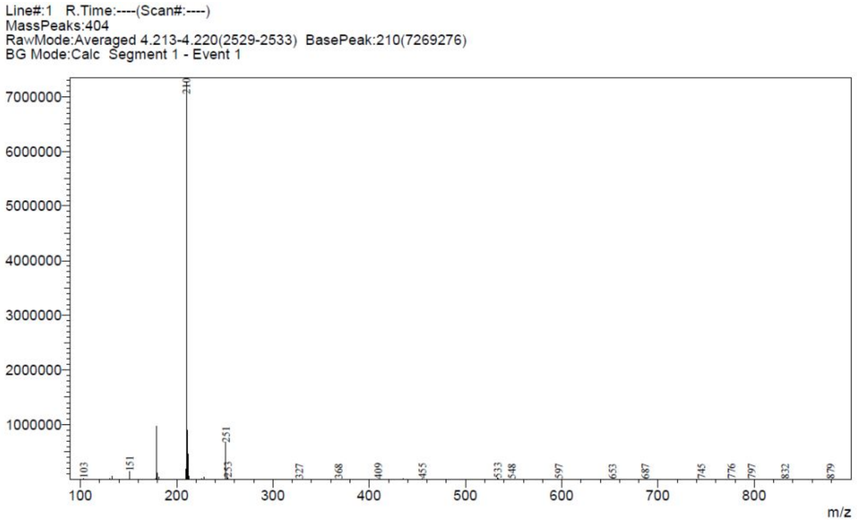

Supplemental Figure 10: LC-MS of SDMA (*m/z* 210.0947), purity SDMA oxalate = 99.9%

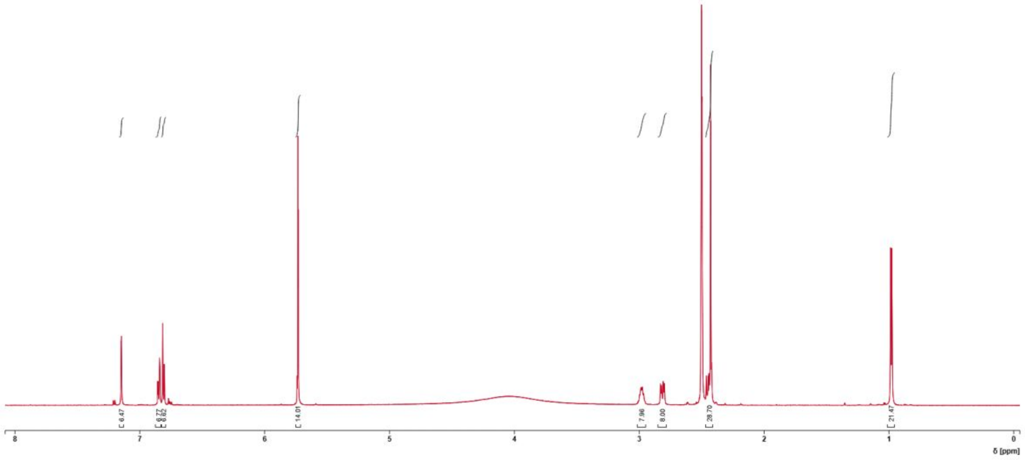

Supplemental Figure 11: ^{1}H-NMR-Spectra (600MHz, d^6-DMSO, 303K) of SDMA. δ (ppm) = 7.15 (d, 1H, H_{arom}); 6.86 (d, 1H, H_{arom}); 6.81 (d, 1H, H_{arom}); 5.72 (s, 2H, $H_{methylen}$); 2.98 (m, 1H, $H_{methylen}$); 2.81 (dd, 1H, $H_{methylen}$); 2.46 (dd, 1H, $H_{methylen}$); 2.42 (s, 3H, N-CH_3); 1.05 (d, 3H, H_{methyl})

1.3 Cell Culture

Stable mono- or polyclonal human embryonic kidney cells (HEK293) cell lines were used as described prior (1). HEK 293 T cells (ATCC; RRID:CVCL_0063) were used for 5-HT_2 Gq protein dissociation assays. Stable cell lines were cultured at 37°C in a humidified atmosphere with 5% CO_2, using Dulbecco's modified Eagle's medium (DMEM) supplemented with 10% fetal bovine serum, 1 U/mL penicillin, 1 µg/mL streptomycin, and 50 µg/mL Geneticin for selection pressure maintenance.

Pheochromocytoma cells (PC12) were cultured in collagen-coated dishes using DMEM supplemented with heat-inactivated 5% fetal bovine serum, 10% horse serum, 10 mM HEPES, 2 mM glutamine, 25 U/mL penicillin, and 25 µg/mL streptomycin (maintenance medium). The cells were incubated at 37°C in a humidified atmosphere with 5% CO_2. For cell differentiation, cells were seeded at a density of 250,000 cells per well in 96-well plates in maintenance medium. After 24 hours, the medium was replaced with a differentiation medium containing 50 ng/mL nerve growth factor. Neurite outgrowth was observed within 24 hours.

1.4 Cell viability (PC12; WST-8 assay)

Treatments with varying concentrations of the drug in DMEM were administered 48 hours after cell differentiation. Each well received 10 µL of the tested drug, in triplicate, and the cells were incubated for 24 hours at 37°C in a humidified atmosphere with 5% CO_2. Following this incubation, the medium was removed and immediately replaced with maintenance medium. Subsequently, 10 µL of CCK-8 Cell Counting Kit, which is based on WST-8 (2-(2-methoxy-4-nitrophenyl)-3-(4-nitrophenyl)-5-(2,4-disulfophenyl)-2H-tetrazolium, monosodium salt), was added to each well. The plates were then incubated for 2 hours at 37°C in a humidified 5% CO_2 atmosphere. Optical density was measured at 450 nm using a microplate reader. Data was statistically evaluated in GraphPad Prism 10.3.1 by One-way ANOVA followed by Tukey's multiple comparisons test (Supplemental Table 8-11).

1.5 Confocal Microscopy

The membrane expression of the expressed transporters was evaluated by confocal microscopy as described in (2). In brief, cells are seeded poly-d-lysine-coated 35 mm glass-bottom dishes 24 hours prior to imaging. On the day of the experiment, cells are incubated with the SOI at IC_{50} concentration or vehicle (KHB) for 60 min and subsequently counter-stained with 0.4% Trypan blue for 4 minutes to visualize the plasma membrane. The fluorescently tagged transporter was subsequently visualized by confocal microscopy using a Nikon laser scanning confocal microscope system (Nikon A1R+ system on an inverted Nikon Ti-E microscope) equipped with a 60x Nikon (NA 1.4) oil immersion objective. The experiment was performed in three replicates, imaging each replicate in five different field-of-views at an excitation wavelength of 488 nm for YFP and 561 nm for Trypan blue. Each image was analyzed using Fiji based on ImageJ2 (3) tracing the cell membrane according to the Trypan blue staining and measuring the mean intensity of the YFP signal in the region of interest. Data was statistically evaluated

in GraphPad Prism 10.3.1 by One-way ANOVA followed by Dunnett's multiple comparisons test (Supplemental Table 12-14).

1.6 Hepatic Metabolism: LC-HRMS/MS conditions

According to previous published procedures analyses were performed using a Thermo Fisher Scientific (TF, Dreieich, Germany) Dionex UltiMate 3000 RS LC system comprising a degasser, a quaternary pump, and an HTC PAL autosampler (CTC Analytics AG, Zwingen, Switzerland), coupled with a TF Q Exactive equipped with a heated electrospray ionization (HESI)-II source (4,5). A TF Accucore Phenyl-Hexyl column (100 mm x 2.1 mm, 2.6 µm) was used for gradient elution at 40°C in accordance with previous publications (4–6). The mobile phase consisted of 2 mM aqueous ammonium formate plus formic acid (0.1%, *v/v*, pH 3, eluent A) and 2 mM ammonium formate solution with acetonitrile:methanol (1:1, *v/v*), water (1%, *v/v*) and formic acid (0.1%, *v/v*, eluent B). The following gradient settings were used: 0-1 min hold 1% B, 1-10 min to 99% B, 10-11.5 min hold 99% B, and 11.5-13.5 min hold 1% B. The flow rate was kept at 500 µl/min from 0-10 min and 800 µl/min from 10-13.5 min. Injection volume was set to 10 µl for all samples. HESI-II source conditions were as follows: ionization mode, positive; sheath gas, 60 AU; auxiliary gas, 10 AU; sweep gas, 0 AU; spray voltage, 4.0 kV; heater temperature 320°C; ion transfer capillary temperature, 320°C; and S-lens RF level, 60.0. Mass spectrometry was performed using full scan and subsequently data-dependent acquisition (DDA) with priority to mass-to-charge ratios (*m/z*) of parent compounds and their expected metabolites. The settings for full scan data acquisition were as follows: resolution 35,000 FWHM at *m/z* 200; microscan, 1; automatic gain control (AGC) target, 1e6; maximum injection time, 120 ms; scan range, *m/z* 50-750; spectrum data type; centroid. Settings for DDA mode with an inclusion list containing the monoisotopic masses of SDMA and SDA and their expected metabolites were as follows: resolution, 17,500 FWHM at *m/z* 200; microscans,1; isolation window, *m/z* 1.0; loop count, 5; AGC target, 2e5; maximum IT, 250 ms; dynamic exclusion, 5 seconds; option “pick others” enabled; high collision dissociation cell with stepped normalized collision energy, 17.5, 35.0, 52.5; exclude isotopes, on; spectrum data type, profile; and underfill ratio, 1%. The inclusion list contained *m/z* values of likely formed metabolites such as hydroxy or dealkyl- metabolites (phase I) as well as sulfates or glucuronides (phase II), and combinations of them. ChemSketch 2012 12.01 (ACD/Labs, Toronto Canada) was used to draw structures of hypothetical metabolites and to calculate exact masses. TF Xcalibur software version 4.5.474.0 was used for data handling.

2 Supplementary Results

2.1 Superfusion Release Assays: Control Substances

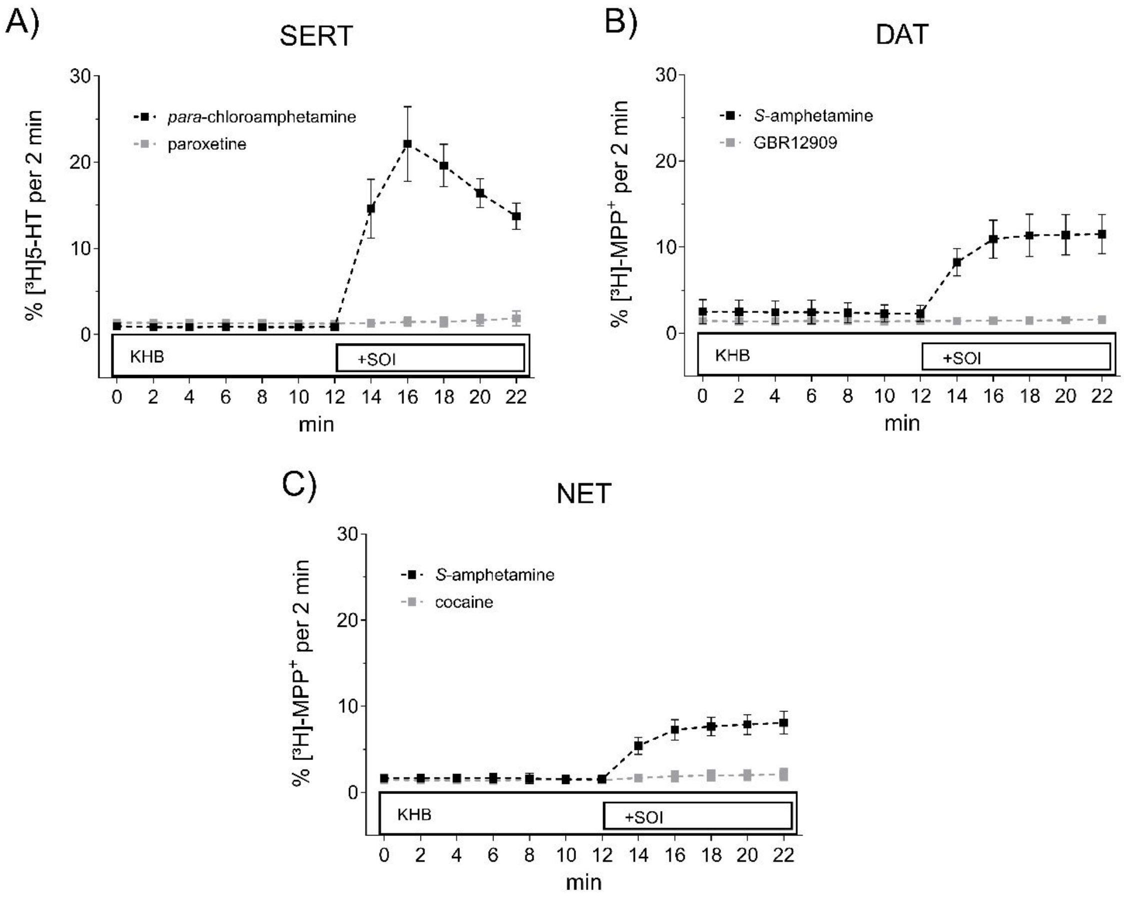

Supplemental Figure 12: Transporter-mediated time-dependent release of [^{3}H]5-HT (SERT) efflux or [^{3}H]5-MPP$^+$ (DAT, NET) elicited by addition of using known releasers and blockers at (A) SERT, (B) DAT and (C) NET. SERT: para-chloroamphetamine 10 µM, paroxetine 0.5 µM. DAT: *S*-amphetamine 10 µM, GBR12909 0.5 µM. NET: S-amphetamine 10 µM, cocaine: 0.5 µM. Data is represented as mean ± standard deviation from three independent experiments (N=3).

2.2 5-HT receptors statistical analysis

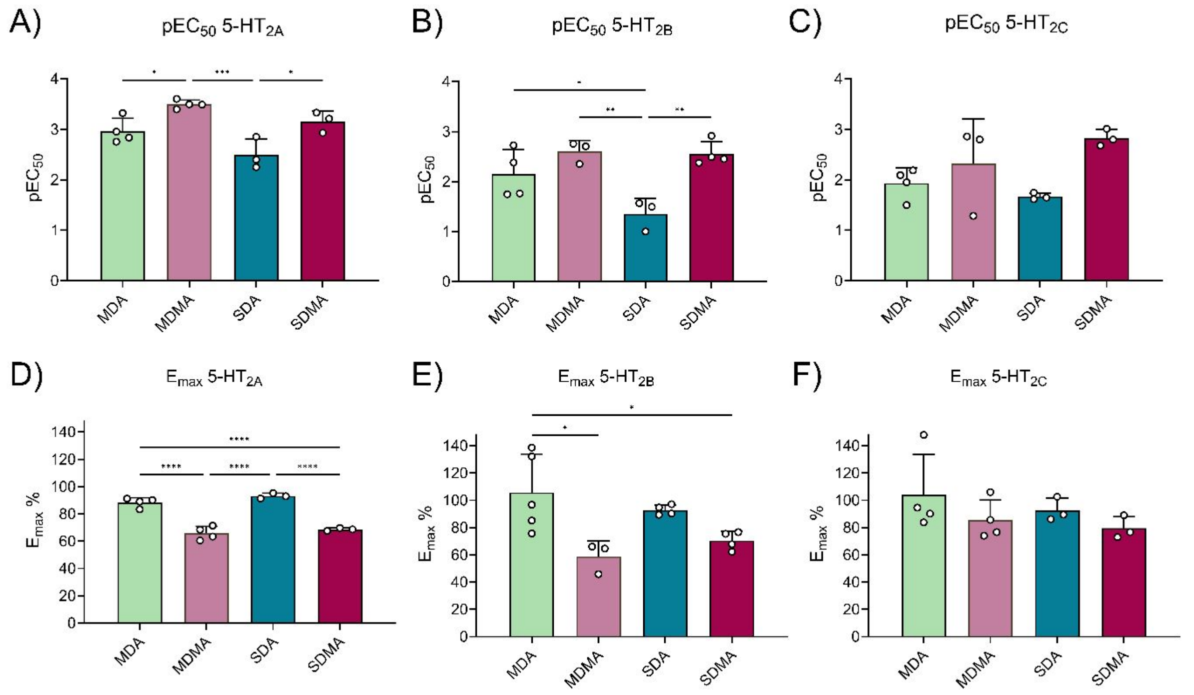

Supplemental Figure 13: Statistical analysis of 5-HT_2 receptor pharmacological responses. pEC_{50} (A–C) values and Emax values (D–F) were compared using one-way ANOVA followed by Tukey's post hoc test. Data are presented as mean ± SD. * p < 0.05, ** p < 0.01, *** p < 0.001, **** p < 0.0001.

Supplemental Table 1: 5-HT_{2A} receptor pEC_{50} statistics (One-way ANOVA) - ANOVA summary

F	12.02
P value	0.0012
P value summary	**
Significant diff. among means (P < 0.05)?	Yes
R squared	0.7829

Supplemental Table 2: 5-HT_{2B} receptor pEC_{50} statistics (One-way ANOVA) - ANOVA summary

F	9.037
P value	0.0034
P value summary	**
Significant diff. among means (P < 0.05)?	Yes
R squared	0.7305

Supplemental Table 3: 5-HT_{2C} receptor pEC_{50} statistics (One-way ANOVA) - ANOVA summary

F	3.625
P value	0.0580
P value summary	ns
Significant diff. among means (P < 0.05)?	No
R squared	0.5471

Supplemental Table 4: 5-HT_{2A} receptor E_{max} statistics (One-way ANOVA) - ANOVA summary

F	54.91
P value	<0.0001
P value summary	****
Significant diff. among means (P < 0.05)?	Yes
R squared	0.9428

Supplemental Table 5: 5-HT_{2B} receptor E_{max} statistics (One-way ANOVA) - ANOVA summary

F	5.830
P value	0.0107
P value summary	*
Significant diff. among means (P < 0.05)?	Yes
R squared	0.5931

Supplemental Table 6: 5-HT_{2C} receptor E_{max} statistics (One-way ANOVA) - ANOVA summary

F	1.138
P value	0.3801
P value summary	ns
Significant diff. among means (P < 0.05)?	No
R squared	0.2546

2.3 OCT Uptake Inhibition

SDMA and SDA interact identically with OCT1 and OCT2 as MDMA and MDA

OCTs significantly influence drug disposition and metabolism by mediating the cellular uptake and elimination of drugs, affecting their bioavailability, efficacy, and toxicity (7). Additionally, they play a key role in maintaining monoaminergic homeostasis by sequestering endogenous substrates. OCTs are expressed in peripheral organs, OCT1 predominately in the liver and OCT2 mainly in the kidneys, and both at a lower level in the brain (8). MDMA and previously analyzed analogues (9,10) showed potent inhibition in the micromolar range of OCT1 and OCT2 with some variation among them, but no or very low inhibition at OCT3. We opted to therefore analyze the potential to inhibit substrate uptake through OCT1 and OCT2, resulting in a very similar interaction profile, as MDA, MDMA, SDA and SDMA all showed a nearly identical IC_{50} at both transporters (Table 1); concentration-response curves are depicted in Supplemental Figure 14.

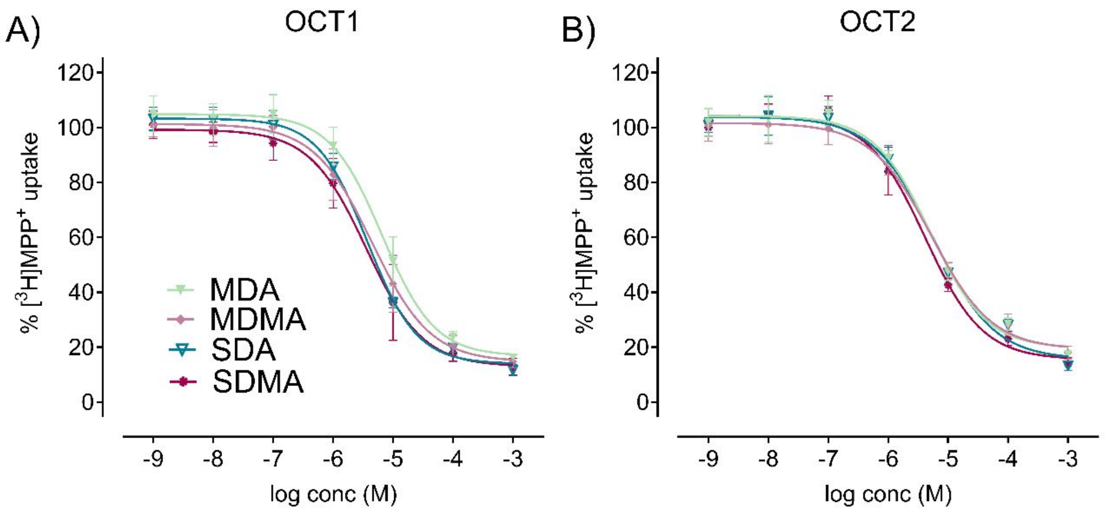

Supplemental Figure 14: Uptake inhibition assays at (A) human organic cation transporter 1 (OCT1), (B) OCT2. Curves were fitted with a sigmoidal dose–response curve to obtain half-maximal inhibitory concentration (IC_{50}). Individual data points are represented with mean ± standard deviation (SD) from three independent experiments, each performed in triplicate (See Table 1, Main Manuscript)

2.4 Molecular docking

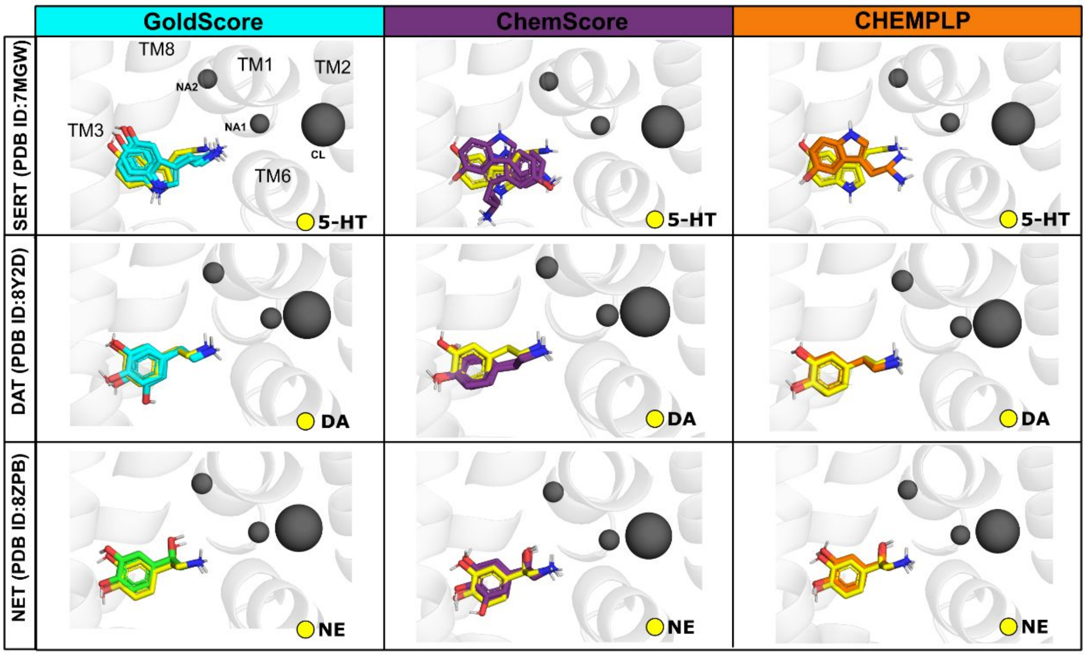

Supplemental Figure 15: Top three docking poses obtained during re-docking. Each row shows the superposition of the first three docking poses ranked using three different scoring functions (GoldSore, CHEMPLP, and ChemScore) for each protein (SERT, DAT, and NET). The cognates are colored yellow, while the predicted binding poses are colored cyan, purple, or orange depending on the scoring function used.

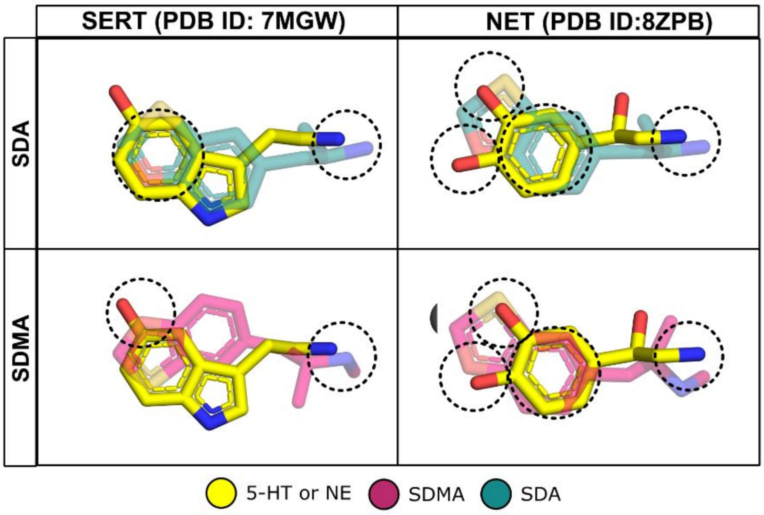

Supplemental Figure 16: The best docking pose for cluster 2 of SDA and SDMA in SERT and NET proteins. Each row represents the superposition of SDA and SDMA with serotonin or norepinephrine. The dashed circles represent the cognate's functional groups that are mimicked in the compound structure. Hydrogen atoms are not displayed.

2.5 Hepatic Metabolism

In total, four phase I and four phase II metabolites of SDMA and three phase I and three phase II metabolites of SDA were tentatively identified in pHLM and/or pHLS9 incubations. In the following section, only exact masses will be used for the characterization of parent compounds and their respective metabolites. High abundant but less characteristic fragmentation ions (FI) of SDMA (precursor ion, PI, at *m/z* 210.0947) as well as SDA (PI at *m/z* 196.0791) were FIs at *m/z* 179.0531 and *m/z* 151.0218.

One of the most abundant metabolites of SDMA was SDMA-M1 (PI *m/z* 196.0791), which originated from *N*-dealkylation. *N*-demethylation is indicated by the absence of FI at *m/z* 58.0657 (C_3H_8N) in comparison to the parent compound. SDMA-M2 (PI at *m/z* 226.0896) was identified as aliphatic hydroxylation by the absence of FI at *m/z* 151.0218 (C_8H_7OS) compared to parent compound. Additionally, the absence of the FI at *m/z* 58.0657 (C_3H_8N), suggesting hydroxylation on the alkyl side chain and not on the ring system. *N*-oxygenation lead to the formation of SDMA-M3 (PI at *m/z* 226.0896) and was identified by the occurrence of the fragment ion at *m/z* 74.0606 (C_3H_8ON). The remaining fragments revealed an unmodified ring system. Additionally, the retention time compared to parent compound supported the theory, since hydroxyl amines are less hydrophilic and therefore, elute later using reversed-phase column, which is in accordance with similar observations concerning hydroxyl amines (11). The SDMA metabolite SDMA-M4 (PI at *m/z* 198.0947) emerged from demethylenation which is indicated by a shift of *m/z* 151.0218 to *m/z* 139.0218 and *m/z* 179.0531 to *m/z* 167.0531, in comparison to the parent compound.

The SDA metabolite SDA-M1 (PI at *m/z* 212.0740) was identified as aliphatic hydroxylation. The FIs of MS^2 spectrum correspond to the SDMA-M2 metabolite. SDA-M2 (PI at *m/z* 212.0740) emerged from *N*-oxygenation. Its MS^2 spectrum is similar to that of the parent compound and the higher retention time compared to parent compound supports this inference. Similar to SDMA, demethylenation was found for SDA and led to the formation of the metabolite SDA-M3 (PI at *m/z* 184.0791). Again, the spectrum matches that of the corresponding SDMA metabolite.

The two demethylenyl-methylation isomers of SDMA SDMA-M5 and SDMA-M6 (PI at *m/z* 212.1104) were formed by demethylenation followed by methylation either on oxygen (SDMA-M5) or sulfur (SDMA-M6), which were represented by the prominent FI's at *m/z* 181.0687 and 153.0374. Both isomers were distinguishable from each other by different RT. Since the electronegativity of oxygen is higher than of sulfur, it could be assumed that methylation at oxygen results in an earlier elution using reversed-phase chromatography. SDMA-M7 (PI at *m/z* 278.0515) stemmed from SDMA-M4 through sulfation of the hydroxy group and their MS^2 spectra were in accordance with each other. *N*-Acetylation after *N*-demethylation led to the formation of SDMA-M8 (PI at *m/z* 238.0896). The spectrum was in accordance with those of SDMA-M1 and also the retention time indicated the *N*-acetylation.

For SDA, two demethylenyl-methylation isomers SDA-M4 and SDA-M5 (PI at *m/z* 198.0947) were also formed. As already described for the SDMA metabolites SDMA-M5 and SDMA-M6, the MS^2 spectra of

the two isomers were in accordance with them. SDA-M6 (PI at m/z 238.0896) was identified as *N*-acetylation. The FIs of MS^2 spectrum and the RT correspond to them of SDMA-M8.

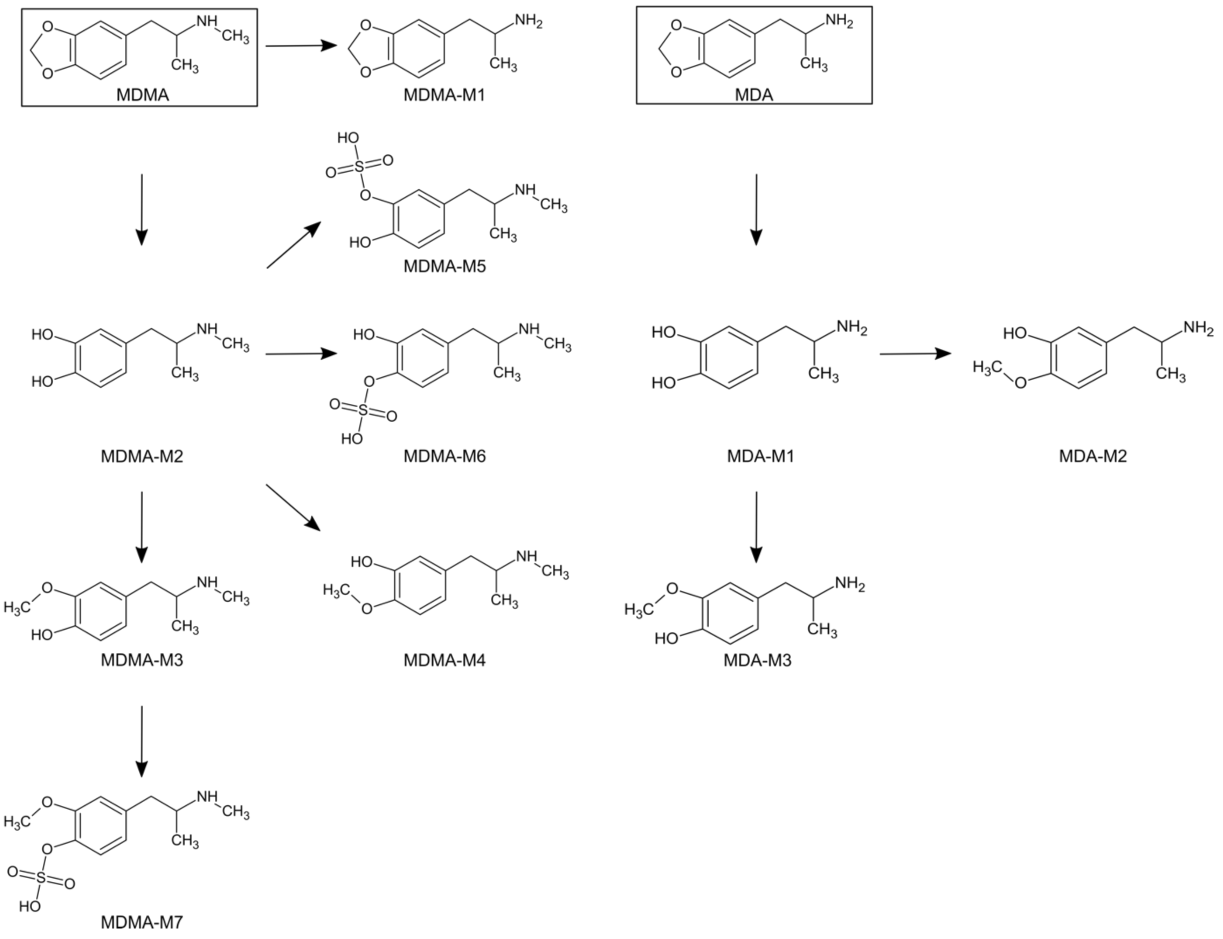

Supplemental Figure 17: Metabolic pathways of MDMA and MDA reported in literature in incubations with pooled human liver microsomes (pHLM) and/or S9 fraction. (12–15)

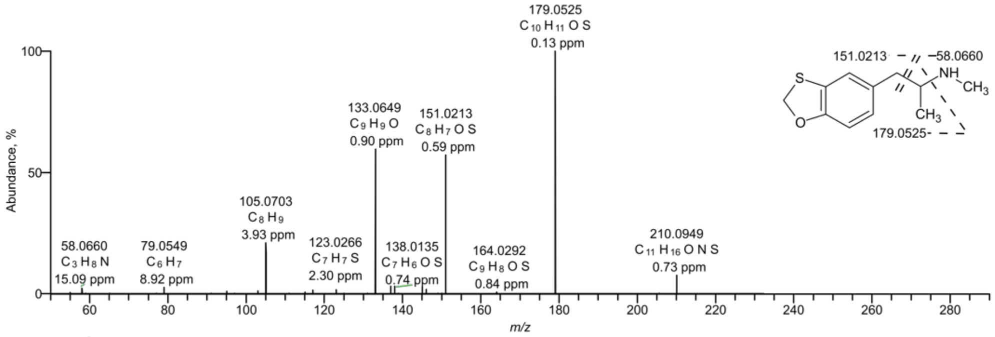

SDMA, MS^2 at *m/z* 210.0947, RT 5.5 min

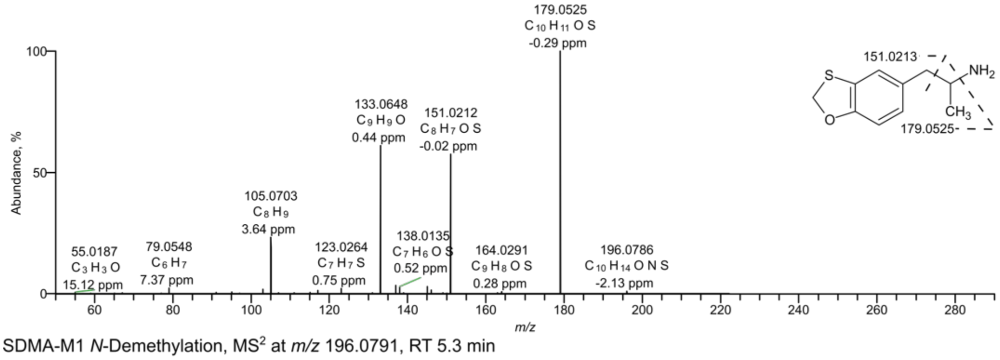

SDMA-M1 *N*-Demethylation, MS^2 at *m/z* 196.0791, RT 5.3 min

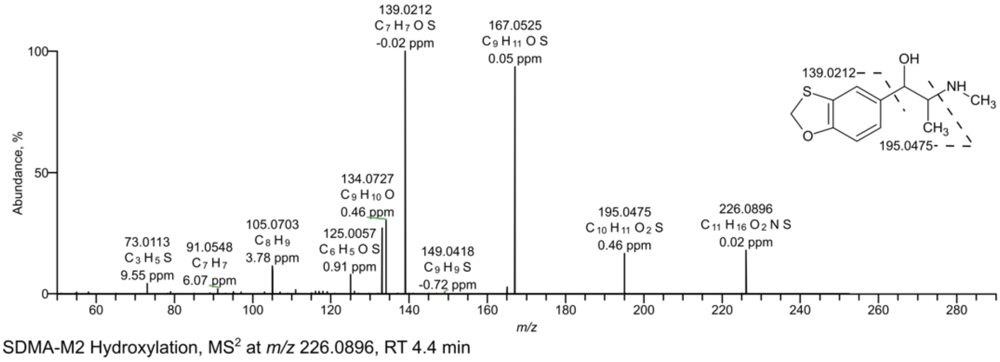

SDMA-M2 Hydroxylation, MS^2 at *m/z* 226.0896, RT 4.4 min

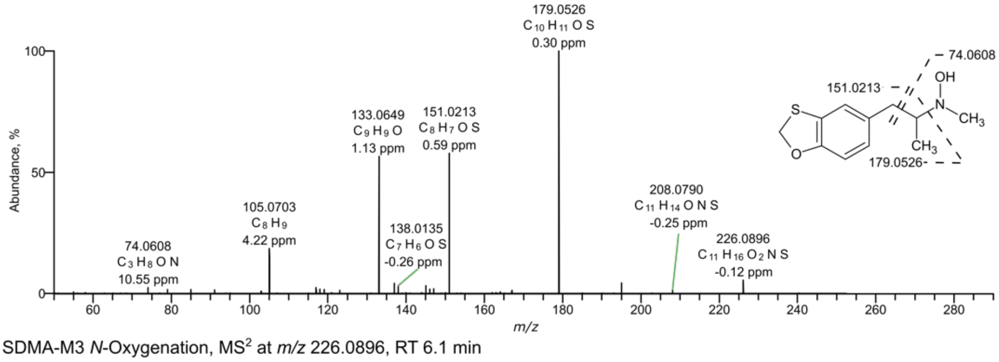

SDMA-M3 *N*-Oxygenation, MS^2 at *m/z* 226.0896, RT 6.1 min

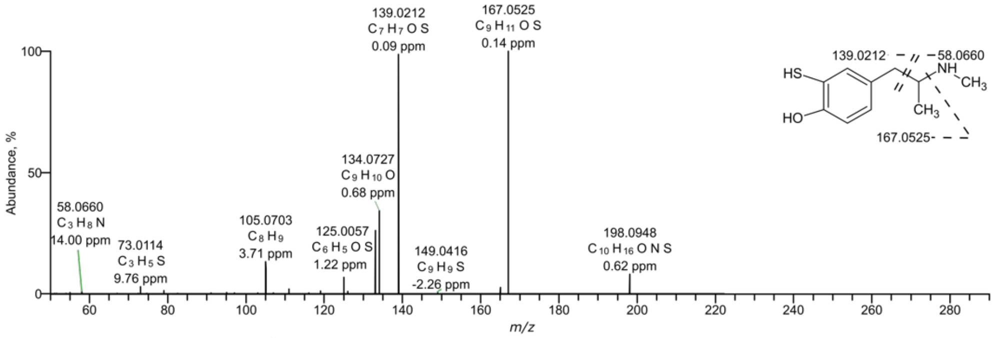

SDMA-M4 Demethylenylation, MS^2 at *m/z* 198.0947, RT 4.3 min

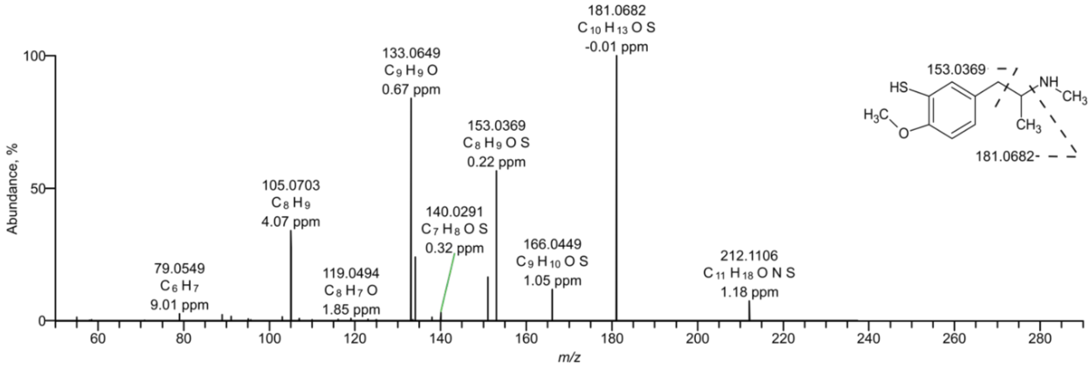

SDMA-M5 Demethylenylation + *O*-methylation, MS^2 at *m/z* 212.1103, RT 4.7 min

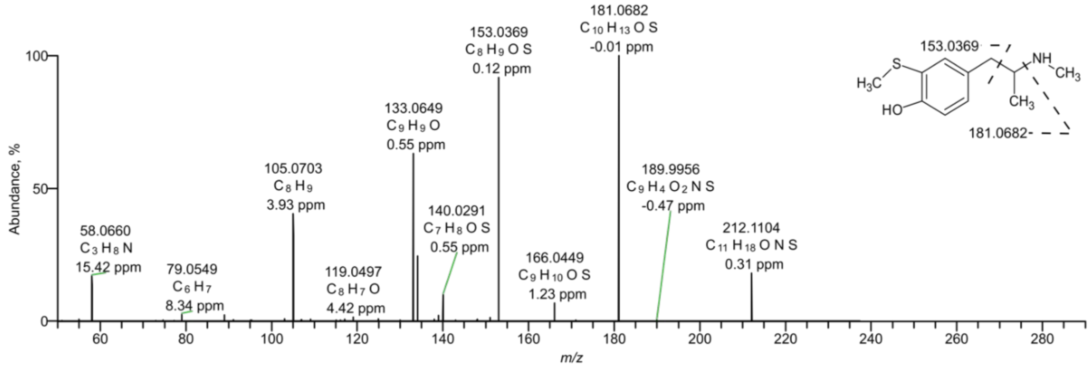

SDMA-M6 Demethylenylation + *S*-methylation, MS^2 at *m/z* 212.1103, RT 5.1 min

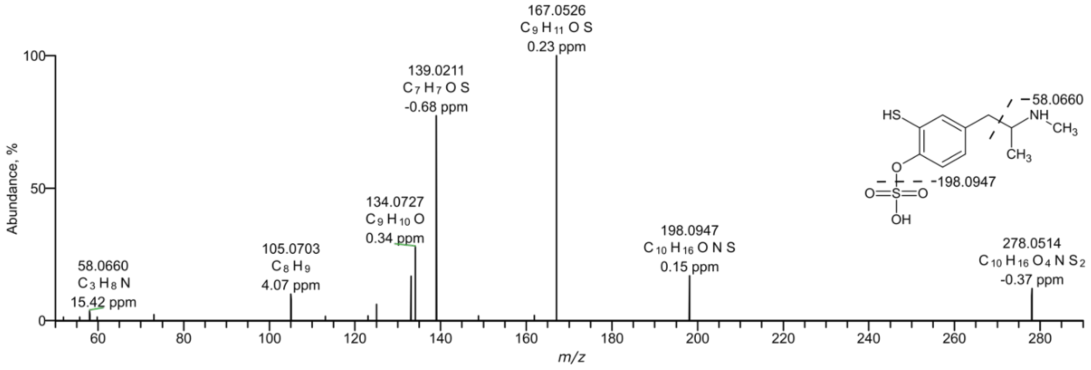

SDMA-M7 Demethylenylation + sulfation, MS^2 at *m/z* 278.0515, RT 4.2 min

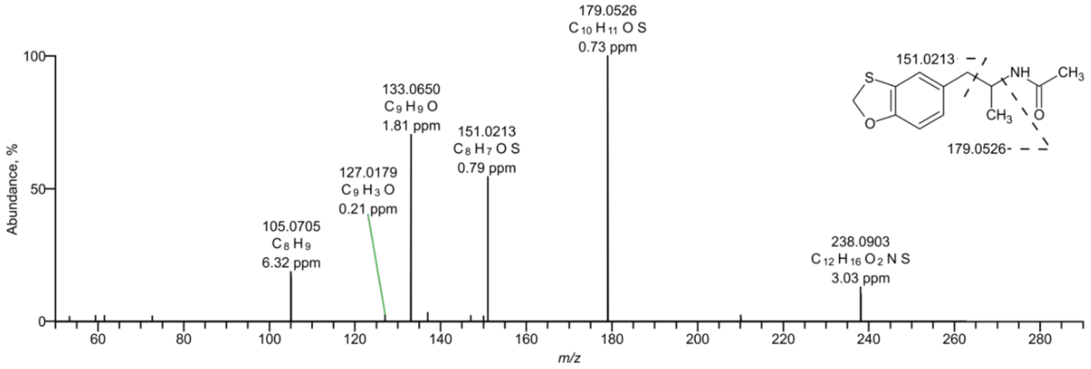

SDMA-M8 *N*-Demethylation + *N*-acetylation, MS^2 at *m/z* 238.0896, RT 7.6 min

Supplemental Figure 18: LC-HRMS/MS spectra of SDMA and its metabolites identified in pooled human liver microsomes or S9 fraction. Metabolite-IDs correspond to Table 7. Fragments with measured mass, calculated elemental formular, and mass error value in parts per million (ppm).

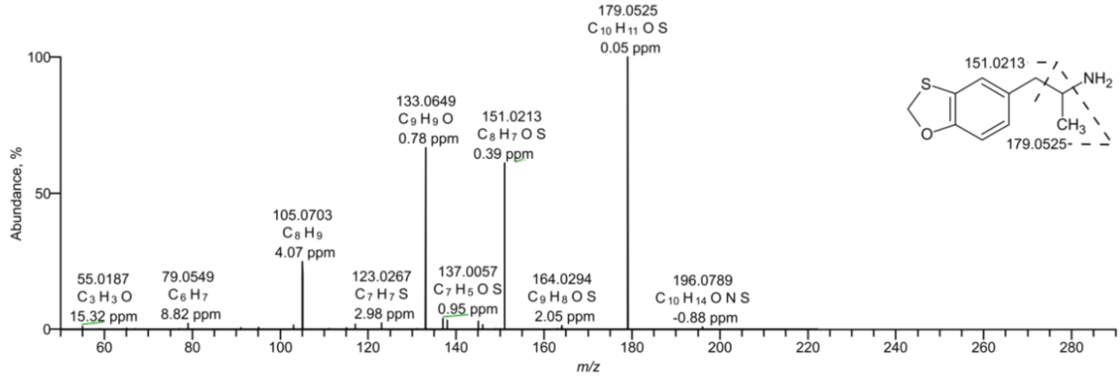

SDA, MS² at *m/z* 196.0791, RT 5.3 min

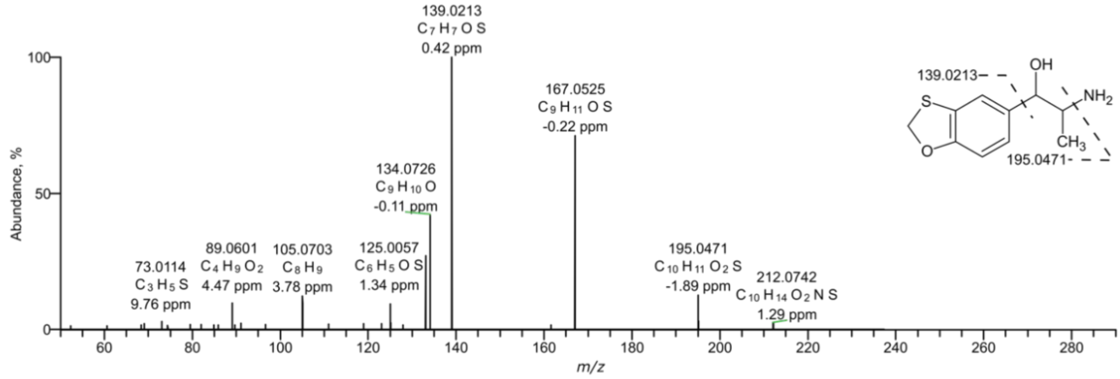

SDA-M1 Hydroxylation, MS² at *m/z* 212.0740, RT 4.2 min

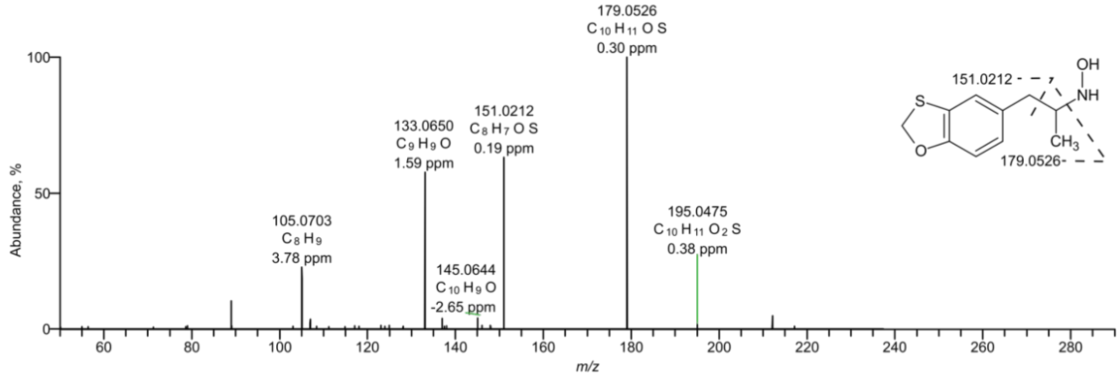

SDA-M2 *N*-Oxygenation, MS² at *m/z* 212.0740, RT 5.8 min

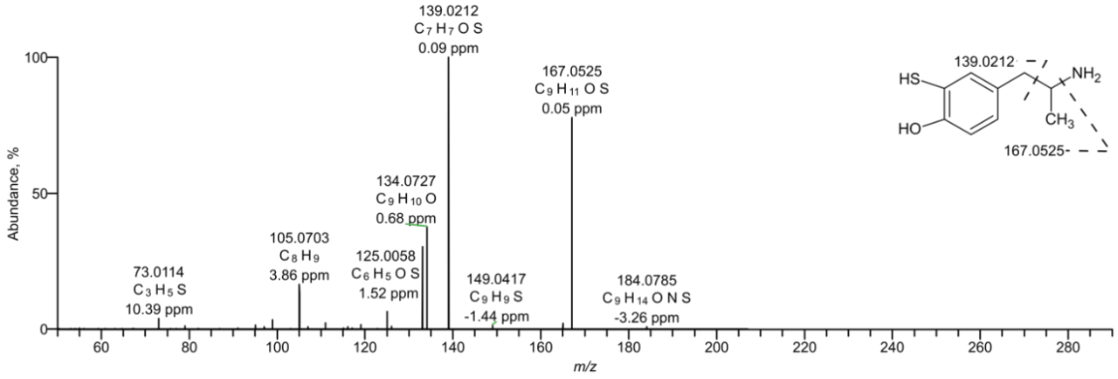

SDA-M3 Demethylenylation, MS² at *m/z* 184.0794, RT 4.0 min

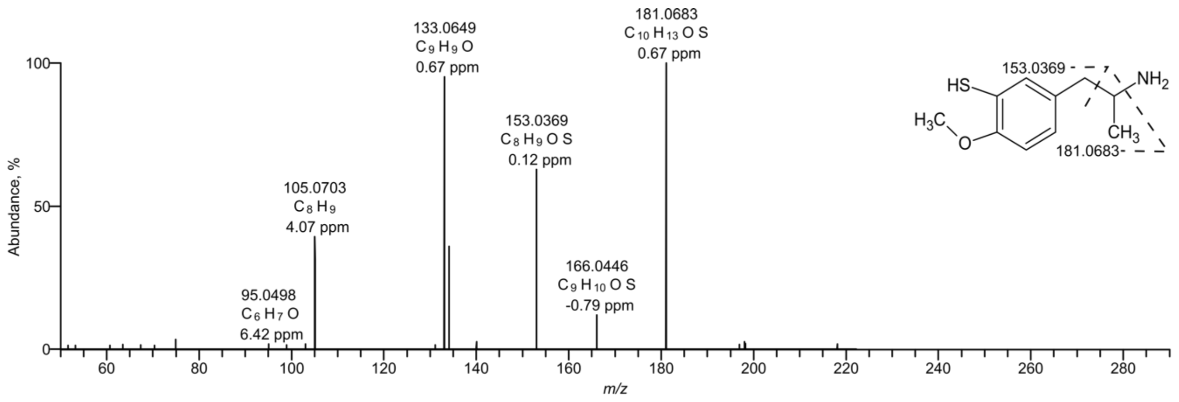

SDA-M4 Demethylenylation + *O*-methylation, MS^2 at *m/z* 198.0947, RT 4.6 min

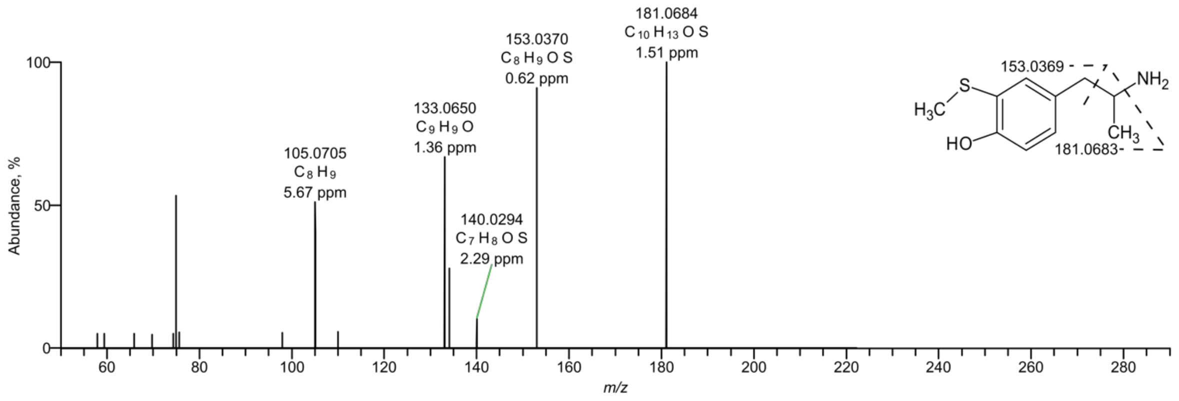

SDA-M5 Demethylenylation + *S*-methylation, MS^2 at *m/z* 198.0947, RT 5.0 min

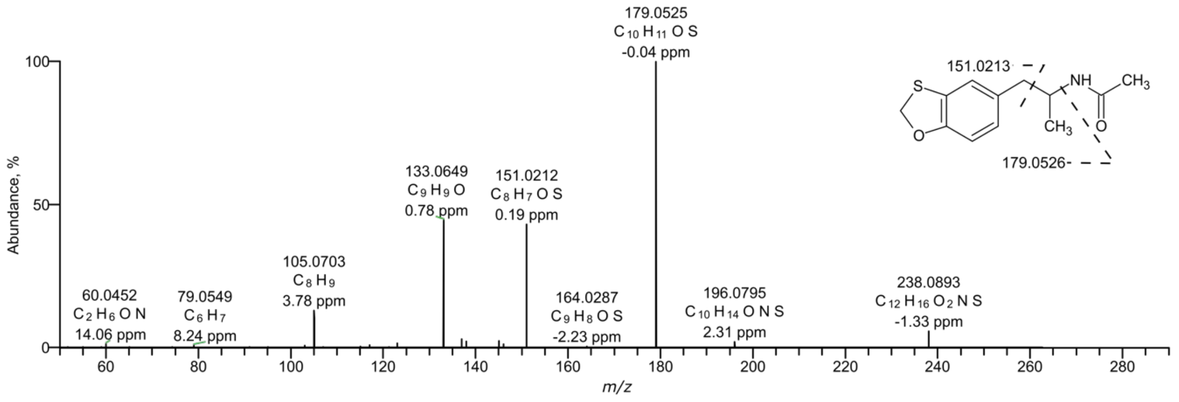

SDA-M6 *N*-Acetylation, MS^2 at *m/z* 238.0896, RT 7.6 min

Supplemental Figure 19: LC-HRMS/MS spectra of SDA and its metabolites identified in pooled human liver microsomes or S9 fraction. Metabolite-IDs correspond to Table 7. Fragments with measured mass, calculated elemental formular, and mass error value in parts per million (ppm).

Supplemental Table 7: Metabolic stability of SDMA, SDA, MDMA, and MDA in pooled human liver microsomes (pHLM) incubations expressed as in vitro half-life ($t_{1/2}$), calculated microsomal intrinsic clearance ($CL_{int, micr}$), and intrinsic clearance (CL_{int}). *, literature data (9).

Compound	$t_{1/2}$ (min)	$CL_{int, micr}$ (mL/min/mg)	CL_{int} (mL/min/kg)
SDMA	71	0.0098	8.4
SDA	61	0.0114	9.7
MDMA*	>150	-	-
MDA	>150	-	-

2.6 Cytotoxicity assays in differentiated PC12 cells and SERT, DAT and NET expressing HEK cells

SDA and SDMA display minimal cytotoxicity

The cytotoxicity potential of MDMA analogues has been examined with NGF-differentiated PC12 cells and HEK293 cells expressing the transporter of interest. Neither MDA and MDMA, nor their novel analogues SDA and SDMA induce cytotoxic effects in the physiologically relevant micromolar range in SERT, DAT or NET expressing HEK293 cells (Supplemental Figure 20A-C). All compounds decreased cell viability in a concentration-dependent manner in PC12 cells; however they were not able to induce cytotoxic effect in the low micromolar range similar as it occurs with MDMA Supplemental Figure 20D. Both MDMA analogues showed cytotoxicity starting in the high micromolar range (800 µM). MDMA showed cytotoxicity starting at 2 mM, although no significant differences are seen at this concentration between compounds.

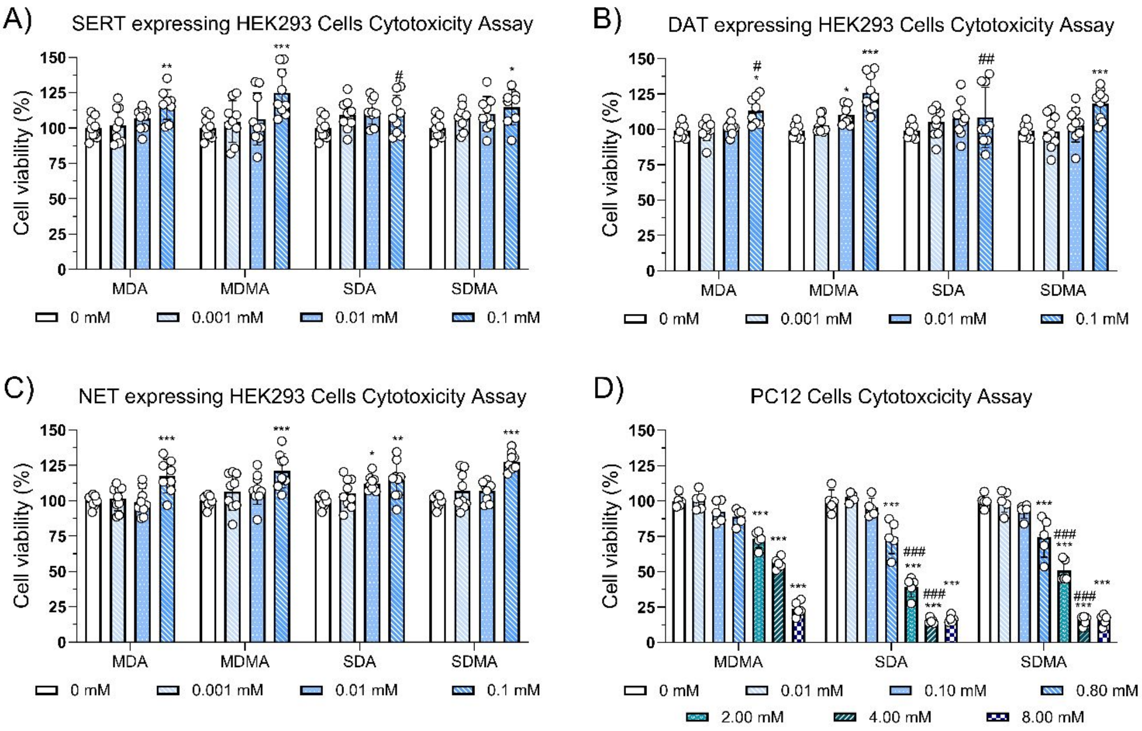

Supplemental Figure 20: Evaluation of the cytotoxicity potential of MDMA analogues in HEK293 cells expressing (A) SERT, (B) DAT or (C) NET using an MTT assay and in (D) NGF-differentiated PC12 cells using the WST-8 assay. Results are expressed as a percentage (%) of cell viability (mean ± SD) of 3-5 experiments carried out on triplicates. Tukey's multiple comparisons tests: * p< 0.05, ** p < 0.01, *** p < 0.001 vs the corresponding control (0 mM) group and # p< 0.05, ## p < 0.01, ### p < 0.001 vs the matching concentration of MDMA.

Supplemental Table 8: SERT expressing HEK293 cytotoxicity assays statistics (Two-way ANOVA) - ANOVA summary:

**ANOVA** table	SS	DF	MS	F (DFn, DFd)	P value
Drug x Concentration	1511	9	167.9	F (9, 127) = 1.244	P=0.2741
Drug	134.4	3	44.80	F (3, 127) = 0.3320	P=0.8022
Concentration	4935	3	1645	F (3, 127) = 12.19	P<0.0001

Supplemental Table 9: DAT expressing HEK293 cytotoxicity assays statistics (Two -way ANOVA) - ANOVA summary:

**ANOVA** table	SS	DF	MS	F (DFn, DFd)	P value
Drug x Concentration	1401	9	155,6	F (9, 127) = 1.523	P=0.1466
Drug	807.9	3	269,3	F (3, 127) = 2.635	P=0.0526
Concentration	6321	3	2107	F (3, 127) = 20.62	P<0.0001

Supplemental Table 10: NET expressing HEK293 cytotoxicity assays statistics (Two -way ANOVA) - ANOVA summary:

**ANOVA** table	SS	DF	MS	F (DFn, DFd)	P value
Drug x Concentration	1177	9	130.7	F (9, 123) = 1.507	P=0.1525
Drug	649.4	3	216.5	F (3, 123) = 2.496	P=0.0630
Concentration	7603	3	2534	F (3, 123) = 29.22	P<0.0001

Supplemental Table 11: PC12 cytotoxicity assays statistics (Two -way ANOVA) - ANOVA summary:

**ANOVA** table	SS	DF	MS	F (DFn, DFd)	P value
Drug x Concentration	5634	12	469.5	F (12, 84) = 10.39	P<0.0001
Drug	3731	2	1866	F (2, 84) = 41.30	P<0.0001
Concentration	105329	6	17555	F (6, 84) = 388.6	P<0.0001

2.7 Transporter membrane expression

SDA and SDMA display minimal impact on transporter surface expression

Previously, it was reported that psychoactive substances can influence transporter expression on plasma membrane level (2). There has been evidence for increased surface expression for cocaine and increased internalization of transporters in the case of amphetamine (16–20) and MDMA (16,21). The novel analogues did not reveal significant changes of expression levels, except for MDA at DAT, where expression levels slightly increased, as depicted in Supplemental Figure 21.

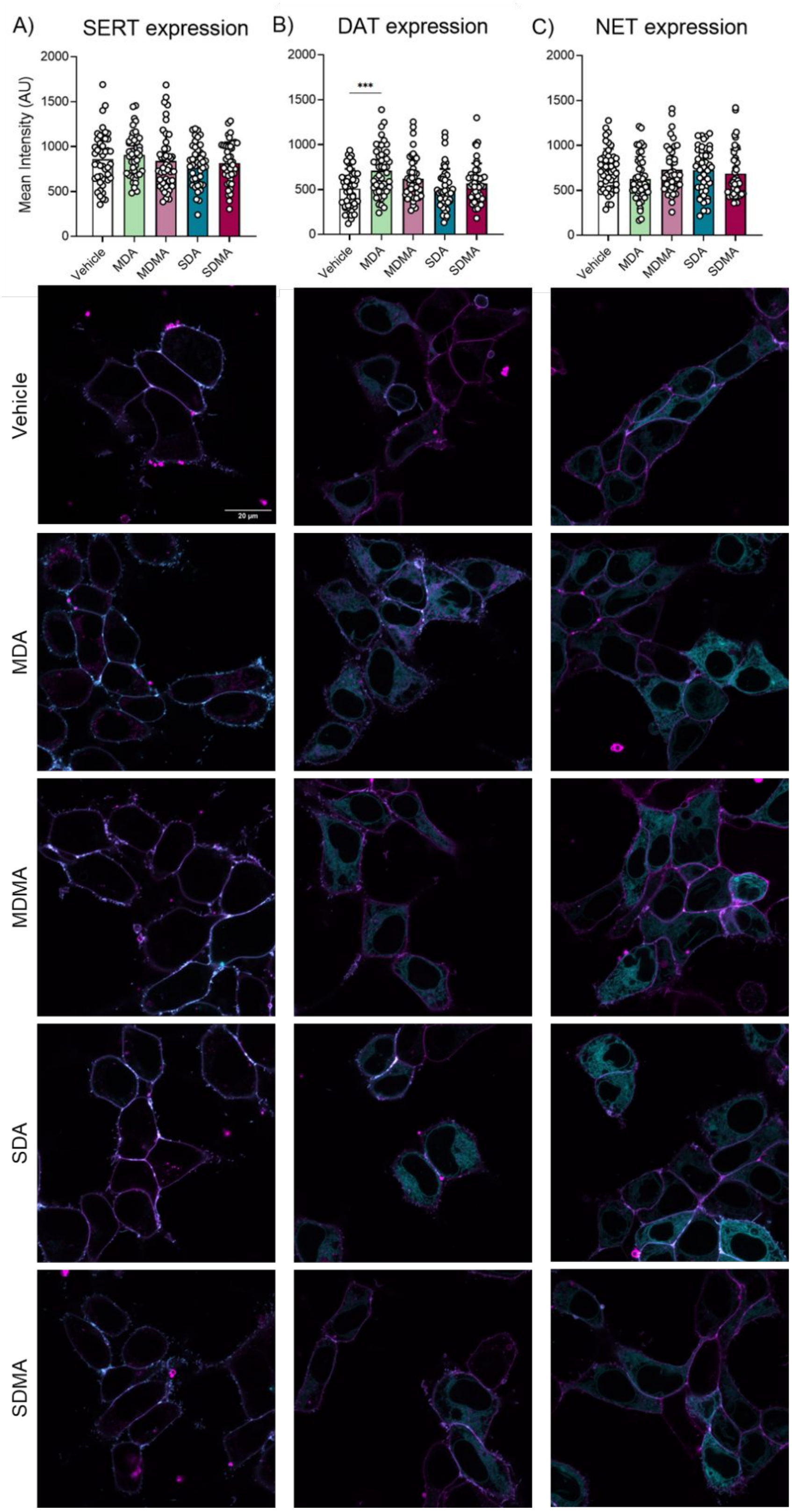

Supplemental Figure 21: Effects of MDA, MDMA, SDA, SDMA on cytotoxicity and transporter expression on plasma membrane level. Data is represented with individual data points and mean ± SD. Statistical analysis was performed by using Dunnett's multiple comparisons test. Scale bar = 20 µm

Supplemental Table 12: SERT expression statistics (One-way ANOVA) - ANOVA summary

F	1.133
P value	0.3418
P value summary	ns
Significant diff. among means (P < 0.05)?	No
R squared	0.02019

Supplemental Table 13: DAT expression statistics (One-way ANOVA) - ANOVA summary

F	6.093
P value	0.0001
P value summary	***
Significant diff. among means (P < 0.05)?	Yes
R squared	0.09852

Supplemental Table 14: NET expression statistics (One-way ANOVA) - ANOVA summary

F	1.438
P value	0.2225
P value summary	ns
Significant diff. among means (P < 0.05)?	No
R squared	0.02547

2.8 *In vivo* behavioral assays

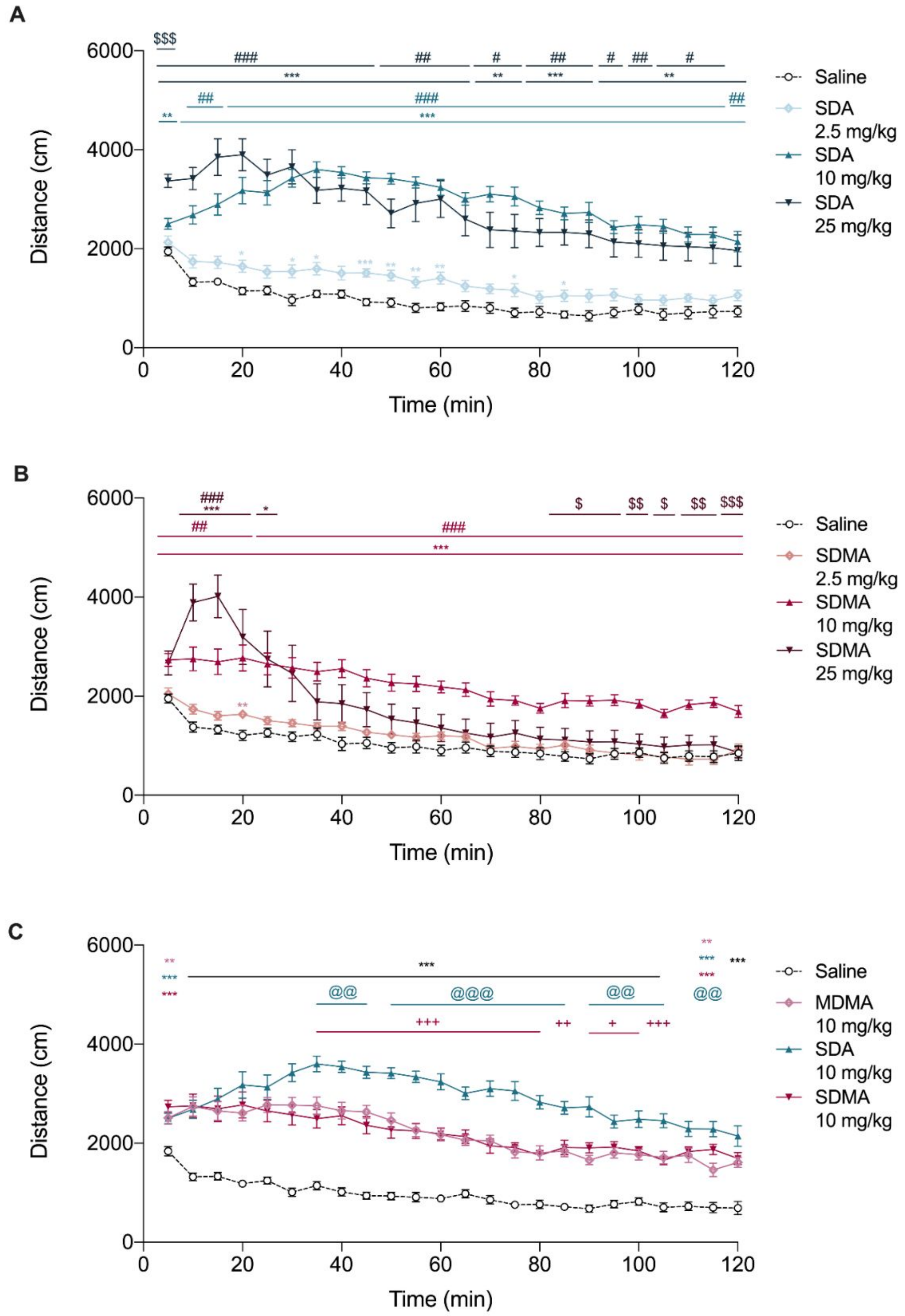

Supplemental Figure 22: Effects of SDA(A),SDMA (B) and its comparison at 10 mg/kg (i.p., calculated as MDMA free base) with MDMA (C)on HLA profile (5 min intervals) in male Swiss CD-1 mice. Data are expressed as mean ± SD of the distance (cm) traveled in 5 min intervals for 2 h. N = 12-14/group. Tukey's multiple-comparison test: * $p < 0.05$, ** $p < 0.01$ and *** $p < 0.001$ vs saline, # $p < 0.05$, ## $p < 0.01$ and ### $p < 0.001$ vs 2.5 mg/kg, $ $p < 0.05$, $$ $p < 0.01$ and $$$ $p < 0.001$ vs 10 mg/kg, @@ $p < 0.01$, @@@ $p < 0.001$ vs MDMA and + $p < 0.05$, ++ $p < 0.01$ and +++ $p < 0.001$ vs SDA.

Supplemental Table 15: HLA total distance SDA statistics (non-parametric test; SDs are significantly different): Kruskal-Wallis test

P value	<0.0001
Exact or approximate P value?	Approximate
P value summary	****
Do the medians vary signif. (P < 0.05)?	Yes
Number of groups	4
Kruskal-Wallis statistic	41.58

Supplemental Table 16: HLA total distance SDMA statistics (non-parametric test; SDs are significantly different): Kruskal-Wallis test

P value	<0.0001
Exact or approximate P value?	Approximate
P value summary	****
Do the medians vary signif. (P < 0.05)?	Yes
Number of groups	4
Kruskal-Wallis statistic	27.87

Supplemental Table 17: HLA total distance at 10 mg/kg statistics (non-parametric test; SDs are significantly different): Kruskal-Wallis test

P value	<0.0001
Exact or approximate P value?	Approximate
P value summary	****
Do the medians vary signif. (P < 0.05)?	Yes
Number of groups	3
Kruskal-Wallis statistic	20.02

Supplemental Table 18: HLA profile SDA statistics (two-way ANOVA of repeated measures)

**ANOVA** table	SS	DF	MS	F (DFn, DFd)	P value
Time x Treatment	75281864	69	1091042	F (69, 1104) = 4.191	P<0.0001
Time	155902782	23	6778382	F (5.086, 244.1) = 26.04	P<0.0001
Treatment	924835835	3	308278612	F (3, 48) = 49.78	P<0.0001
Subject	297270081	48	6193127	F (48, 1104) = 23.79	P<0.0001

Supplemental Table 19: HLA profile SDMA statistics (two-way ANOVA of repeated measures)

**ANOVA** table	SS	DF	MS	F (DFn, DFd)	P value
Time x Treatment	115083887	69	1667882	F (69, 1196) = 6.096	P<0.0001
Time	274927335	23	11953362	F (3.499, 182.0) = 43.69	P<0.0001
Treatment	291505992	3	97168664	F (3, 52) = 14.55	P<0.0001
Subject	347193850	52	6676805	F (52, 1196) = 24.40	P<0.0001

Supplemental Table 20: HLA profile at 10 mg/kg statistics (two-way ANOVA of repeated measures)

**ANOVA** table	SS	DF	MS	F (DFn, DFd)	P value
Time x Treatment	47234088	69	684552	F (69, 1127) = 4.365	P<0.0001
Time	137508740	23	5978641	F (3,742, 183,3) = 38.13	P<0.0001
Treatment	630083755	3	210027918	F (3, 49) = 79.79	P<0.0001
Subject	128976962	49	2632183	F (49, 1127) = 16.79	P<0.0001

Supplemental Table 21: CENTER vs. PERIPHERY SDA statistics (non-parametric test; SDs are significantly different): Kruskal-Wallis test

P value	0.0019
Exact or approximate P value?	Approximate
P value summary	**
Do the medians vary signif. (P < 0.05)?	Yes
Number of groups	4
Kruskal-Wallis statistic	14.85

Supplemental Table 22: CENTER vs. PERIPHERY SDMA statistics (non-parametric test; SDs are significantly different /does not follow normality): Kruskal-Wallis test

P value	0.0542
Exact or approximate P value?	Approximate
P value summary	ns
Do the medians vary signif. (P < 0.05)?	No
Number of groups	4
Kruskal-Wallis statistic	27.87

Supplemental Table 23: CENTER VS. PERIPHERY at 10 mg/kg statistics (One-way ANOVA) - ANOVA summary

F	4.255
P value	0.0095
P value summary	**
Significant diff. among means (P < 0.05)?	Yes
R squared	0.2067

Supplemental Table 24: CPP SDA statistics (non-parametric test; SDs are significantly different): Kruskal-Wallis test

P value	0.0148
Exact or approximate P value?	Approximate
P value summary	*
Do the medians vary signif. (P < 0.05)?	Yes
Number of groups	4
Kruskal-Wallis statistic	10.49

Supplemental Table 25: CPP SDMA statistics (One-way ANOVA) - ANOVA summary

F	1.233
P value	0.3085
P value summary	ns
Significant diff. among means (P < 0.05)?	No
R squared	0.07443

Supplemental Table 26: CPP 10 mg/kg statistics (non-parametric test; SDs are significantly different): Kruskal-Wallis test

P value	0.0413
Exact or approximate P value?	Approximate
P value summary	*
Do the medians vary signif. (P < 0.05)?	Yes
Number of groups	4
Kruskal-Wallis statistic	8.238

Supplemental Table 27: Core body temperature statistics (22±1 ºC) - Mixed-effects model

**Mixed-effects model (22±1 ºC)**	P value	"P value summary"	"F (DFn, DFd)"	"Geisser-Greenhouse's epsilon"
Time x Treatment	<0.0001	****	"F (2.273, 54.55) = 12.82"	0.1263
Time	<0.0001	****	"F (3, 24) = 11.15"	
Treatment	<0.0001	****	"F (54, 432) = 5.283"	

Supplemental Table 28: Core body temperature statistics (27±1 ºC) - Mixed-effects model

**Mixed-effects model (27±1 ºC)**	P value	"P value summary"	"F (DFn, DFd)"	"Geisser-Greenhouse's epsilon"
Time x Treatment	<0.0001	****	"F (2.534, 59.68) = 17.57"	0.1408
Time	<0.0001	****	"F (3, 24) = 20.51"	
Treatment	<0.0001	****	"F (54, 424) = 8.611"	

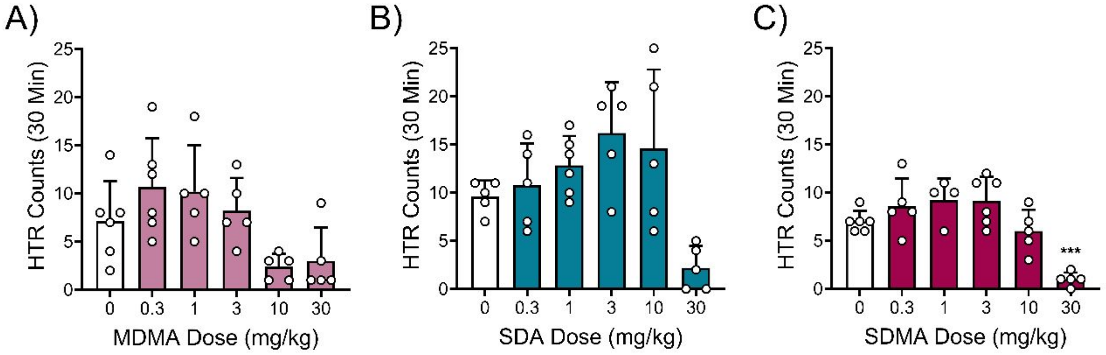

Supplemental Figure 23: Effect of (A) MDMA, (B) SDA and (C) SDMA on the head twitch response (HTR). Doses were calculated based on the respective salt forms of the compounds. Data are presented as means ± SD for the 30-min test session. Statistical analysis was carried out using One-way ANOVA following Dunnett's multiple comparisons test. N=4-6 per group. *** p < 0.001.

Supplemental Table 29: HTR MDMA statistics - (One-way ANOVA) - ANOVA summary

F	4.131
P value	0.0068
P value summary	**
Significant diff. among means (P < 0.05)?	Yes
R squared	0.4427

Supplemental Table 30: HTR SDA statistics - (One-way ANOVA) - ANOVA summary

F	5.782
P value	0.0011
P value summary	**
Significant diff. among means (P < 0.05)?	Yes
R squared	0.5363

Supplemental Table 31: HTR SDMA statistics - (One-way ANOVA) - ANOVA summary

F	11.49
P value	<0.0001
P value summary	****
Significant diff. among means (P < 0.05)?	Yes
R squared	0.6968

3 References

1. Mayer FP, Luf A, Nagy C, Holy M, Schmid R, Freissmuth M, et al. Application of a Combined Approach to Identify New Psychoactive Street Drugs and Decipher Their Mechanisms at Monoamine Transporters. In: Baumann MH, Glennon RA, Wiley JL, editors. Neuropharmacology of New Psychoactive Substances (NPS): The Science Behind the Headlines [Internet]. Cham: Springer International Publishing; 2017 [cited 2024 Sep 16]. p. 333–50. Available from: https://doi.org/10.1007/7854_2016_63

2. Niello M, Sideromenos S, Gradisch R, O´Shea R, Schwazer J, Maier J, et al. Persistent binding at dopamine transporters determines sustained psychostimulant effects. Proc Natl Acad Sci. 2023 Feb 7;120(6):e2114204120.

3. Schindelin J, Arganda-Carreras I, Frise E, Kaynig V, Longair M, Pietzsch T, et al. Fiji: an open-source platform for biological-image analysis. Nat Methods. 2012 Jul;9(7):676–82.

4. Gampfer TM, Schütz V, Schippers P, Rasheed S, Baumann J, Wagmann L, et al. Metabolism and cytotoxicity studies of the two hallucinogens 1cP-LSD and 4-AcO-DET in human liver and zebrafish larvae models using LC-HRMS/MS and a high-content screening assay. J Pharm Biomed Anal. 2024 Aug 1;245:116187.

5. Richter MJ, Wagmann L, Gampfer TM, Brandt SD, Meyer MR. In Vitro Metabolic Fate of the Synthetic Cannabinoid Receptor Agonists QMPSB and QMPCB (SGT-11) Including Isozyme Mapping and Esterase Activity. Metabolites. 2021 Aug 3;11(8):509.

6. Helfer AG, Michely JA, Weber AA, Meyer MR, Maurer HH. Orbitrap technology for comprehensive metabolite-based liquid chromatographic-high resolution-tandem mass spectrometric urine drug screening - exemplified for cardiovascular drugs. Anal Chim Acta. 2015 Sep 3;891:221–33.

7. Koepsell H. Organic Cation Transporters in Health and Disease. Daws LC, editor. Pharmacol Rev. 2020 Jan 1;72(1):253–319.

8. Gorboulev V, Ulzheimer JC, Akhoundova A, Ulzheimer-Teuber I, Karbach U, Quester S, et al. Cloning and Characterization of Two Human Polyspecific Organic Cation Transporters. DNA Cell Biol. 1997 Jul;16(7):871–81.

9. Alberto-Silva AS, Hemmer S, Bock HA, da Silva LA, Scott KR, Kastner N, et al. Bioisosteric analogs of MDMA: Improving the pharmacological profile? J Neurochem. 2024;168(9):2022–42.

10. Angenoorth TJF, Stankovic S, Niello M, Holy M, Brandt SD, Sitte HH, et al. Interaction Profiles of Central Nervous System Active Drugs at Human Organic Cation Transporters 1–3 and Human Plasma Membrane Monoamine Transporter. Int J Mol Sci. 2021 Nov 30;22(23):12995.

11. Manier SK, Felske C, Eckstein N, Meyer MR. The metabolic fate of two new psychoactive substances - 2-aminoindane and N-methyl-2-aminoindane - studied in vitro and in vivo to support drug testing. Drug Test Anal. 2020 Jan;12(1):145–51.

12. Kraemer T, Maurer HH. Toxicokinetics of Amphetamines: Metabolism and Toxicokinetic Data of Designer Drugs, Amphetamine, Methamphetamine, and Their N-Alkyl Derivatives. Ther Drug Monit. 2002 Apr;24(2):277.

13. Maurer HH, Bickeboeller-Friedrich J, Kraemer T, Peters FT. Toxicokinetics and analytical toxicology of amphetamine-derived designer drugs (‘Ecstasy’). Toxicol Lett. 2000 Mar 15;112–113:133–42.

14. Richter LHJ, Flockerzi V, Maurer HH, Meyer MR. Pooled human liver preparations, HepaRG, or HepG2 cell lines for metabolism studies of new psychoactive substances? A study using MDMA, MDBD, butylone, MDPPP, MDPV, MDPB, 5-MAPB, and 5-API as examples. J Pharm Biomed Anal. 2017 Sep 5;143:32–42.

15. Schwaninger AE, Meyer MR, Zapp J, Maurer HH. Sulfation of the 3,4-methylenedioxymethamphetamine (MDMA) metabolites 3,4-dihydroxymethamphetamine (DHMA) and 4-hydroxy-3-methoxymethamphetamine (HMMA) and their capability to inhibit human sulfotransferases. Toxicol Lett. 2011 Apr 25;202(2):120–8.

16. Lau T, Schloss P. Differential regulation of serotonin transporter cell surface expression. Wiley Interdiscip Rev Membr Transp Signal. 2012;1(3):259–68.

17. Little KY, Elmer LW, Zhong H, Scheys JO, Zhang L. Cocaine Induction of Dopamine Transporter Trafficking to the Plasma Membrane. Mol Pharmacol. 2002 Feb 1;61(2):436–45.

18. Saenz J, Yao O, Khezerlou E, Aggarwal M, Zhou X, Barker DJ, et al. Cocaine-regulated trafficking of dopamine transporters in cultured neurons revealed by a pH sensitive reporter. iScience. 2022 Dec 9;26(1):105782.

19. Saunders C, Ferrer JV, Shi L, Chen J, Merrill G, Lamb ME, et al. Amphetamine-induced loss of human dopamine transporter activity: An internalization-dependent and cocaine-sensitive mechanism. Proc Natl Acad Sci. 2000 Jun 6;97(12):6850–5.

20. Wheeler DS, Underhill SM, Stolz DB, Murdoch GH, Thiels E, Romero G, et al. Amphetamine activates Rho GTPase signaling to mediate dopamine transporter internalization and acute behavioral effects of amphetamine. Proc Natl Acad Sci. 2015 Dec 22;112(51):E7138–47.

21. Kivell B, Day D, Bosch P, Schenk S, Miller J. MDMA causes a redistribution of serotonin transporter from the cell surface to the intracellular compartment by a mechanism independent of phospho-p38-mitogen activated protein kinase activation. Neuroscience. 2010 Jun 16;168(1):82–95.
